# Search for gluinos in events with an isolated lepton, jets and missing transverse momentum at $\sqrt{s}$ = 13 Te V with the ATLAS detector

**DOI:** 10.1140/epjc/s10052-016-4397-x

**Published:** 2016-10-21

**Authors:** G. Aad, B. Abbott, J. Abdallah, O. Abdinov, B. Abeloos, R. Aben, M. Abolins, O. S. AbouZeid, H. Abramowicz, H. Abreu, R. Abreu, Y. Abulaiti, B. S. Acharya, L. Adamczyk, D. L. Adams, J. Adelman, S. Adomeit, T. Adye, A. A. Affolder, T. Agatonovic-Jovin, J. Agricola, J. A. Aguilar-Saavedra, S. P. Ahlen, F. Ahmadov, G. Aielli, H. Akerstedt, T. P. A. Åkesson, A. V. Akimov, G. L. Alberghi, J. Albert, S. Albrand, M. J. Alconada Verzini, M. Aleksa, I. N. Aleksandrov, C. Alexa, G. Alexander, T. Alexopoulos, M. Alhroob, G. Alimonti, J. Alison, S. P. Alkire, B. M. M. Allbrooke, B. W. Allen, P. P. Allport, A. Aloisio, A. Alonso, F. Alonso, C. Alpigiani, B. Alvarez Gonzalez, D. Álvarez Piqueras, M. G. Alviggi, B. T. Amadio, K. Amako, Y. Amaral Coutinho, C. Amelung, D. Amidei, S. P. Amor Dos Santos, A. Amorim, S. Amoroso, N. Amram, G. Amundsen, C. Anastopoulos, L. S. Ancu, N. Andari, T. Andeen, C. F. Anders, G. Anders, J. K. Anders, K. J. Anderson, A. Andreazza, V. Andrei, S. Angelidakis, I. Angelozzi, P. Anger, A. Angerami, F. Anghinolfi, A. V. Anisenkov, N. Anjos, A. Annovi, M. Antonelli, A. Antonov, J. Antos, F. Anulli, M. Aoki, L. Aperio Bella, G. Arabidze, Y. Arai, J. P. Araque, A. T. H. Arce, F. A. Arduh, J-F. Arguin, S. Argyropoulos, M. Arik, A. J. Armbruster, L. J. Armitage, O. Arnaez, H. Arnold, M. Arratia, O. Arslan, A. Artamonov, G. Artoni, S. Artz, S. Asai, N. Asbah, A. Ashkenazi, B. Åsman, L. Asquith, K. Assamagan, R. Astalos, M. Atkinson, N. B. Atlay, K. Augsten, G. Avolio, B. Axen, M. K. Ayoub, G. Azuelos, M. A. Baak, A. E. Baas, M. J. Baca, H. Bachacou, K. Bachas, M. Backes, M. Backhaus, P. Bagiacchi, P. Bagnaia, Y. Bai, J. T. Baines, O. K. Baker, E. M. Baldin, P. Balek, T. Balestri, F. Balli, W. K. Balunas, E. Banas, Sw. Banerjee, A. A. E. Bannoura, L. Barak, E. L. Barberio, D. Barberis, M. Barbero, T. Barillari, T. Barklow, N. Barlow, S. L. Barnes, B. M. Barnett, R. M. Barnett, Z. Barnovska, A. Baroncelli, G. Barone, A. J. Barr, L. Barranco Navarro, F. Barreiro, J. Barreiro Guimarães da Costa, R. Bartoldus, A. E. Barton, P. Bartos, A. Basalaev, A. Bassalat, A. Basye, R. L. Bates, S. J. Batista, J. R. Batley, M. Battaglia, M. Bauce, F. Bauer, H. S. Bawa, J. B. Beacham, M. D. Beattie, T. Beau, P. H. Beauchemin, P. Bechtle, H. P. Beck, K. Becker, M. Becker, M. Beckingham, C. Becot, A. J. Beddall, A. Beddall, V. A. Bednyakov, M. Bedognetti, C. P. Bee, L. J. Beemster, T. A. Beermann, M. Begel, J. K. Behr, C. Belanger-Champagne, A. S. Bell, G. Bella, L. Bellagamba, A. Bellerive, M. Bellomo, K. Belotskiy, O. Beltramello, N. L. Belyaev, O. Benary, D. Benchekroun, M. Bender, K. Bendtz, N. Benekos, Y. Benhammou, E. Benhar Noccioli, J. Benitez, J. A. Benitez Garcia, D. P. Benjamin, J. R. Bensinger, S. Bentvelsen, L. Beresford, M. Beretta, D. Berge, E. Bergeaas Kuutmann, N. Berger, F. Berghaus, J. Beringer, S. Berlendis, N. R. Bernard, C. Bernius, F. U. Bernlochner, T. Berry, P. Berta, C. Bertella, G. Bertoli, F. Bertolucci, I. A. Bertram, C. Bertsche, D. Bertsche, G. J. Besjes, O. Bessidskaia Bylund, M. Bessner, N. Besson, C. Betancourt, S. Bethke, A. J. Bevan, W. Bhimji, R. M. Bianchi, L. Bianchini, M. Bianco, O. Biebel, D. Biedermann, R. Bielski, N. V. Biesuz, M. Biglietti, J. Bilbao De Mendizabal, H. Bilokon, M. Bindi, S. Binet, A. Bingul, C. Bini, S. Biondi, D. M. Bjergaard, C. W. Black, J. E. Black, K. M. Black, D. Blackburn, R. E. Blair, J. -B. Blanchard, J. E. Blanco, T. Blazek, I. Bloch, C. Blocker, W. Blum, U. Blumenschein, S. Blunier, G. J. Bobbink, V. S. Bobrovnikov, S. S. Bocchetta, A. Bocci, C. Bock, M. Boehler, D. Boerner, J. A. Bogaerts, D. Bogavac, A. G. Bogdanchikov, C. Bohm, V. Boisvert, T. Bold, V. Boldea, A. S. Boldyrev, M. Bomben, M. Bona, M. Boonekamp, A. Borisov, G. Borissov, J. Bortfeldt, D. Bortoletto, V. Bortolotto, K. Bos, D. Boscherini, M. Bosman, J. D. Bossio Sola, J. Boudreau, J. Bouffard, E. V. Bouhova-Thacker, D. Boumediene, C. Bourdarios, N. Bousson, S. K. Boutle, A. Boveia, J. Boyd, I. R. Boyko, J. Bracinik, A. Brandt, G. Brandt, O. Brandt, U. Bratzler, B. Brau, J. E. Brau, H. M. Braun, W. D. Breaden Madden, K. Brendlinger, A. J. Brennan, L. Brenner, R. Brenner, S. Bressler, T. M. Bristow, D. Britton, D. Britzger, F. M. Brochu, I. Brock, R. Brock, G. Brooijmans, T. Brooks, W. K. Brooks, J. Brosamer, E. Brost, J. H Broughton, P. A. Bruckman de Renstrom, D. Bruncko, R. Bruneliere, A. Bruni, G. Bruni, BH Brunt, M. Bruschi, N. Bruscino, P. Bryant, L. Bryngemark, T. Buanes, Q. Buat, P. Buchholz, A. G. Buckley, I. A. Budagov, F. Buehrer, M. K. Bugge, O. Bulekov, D. Bullock, H. Burckhart, S. Burdin, C. D. Burgard, B. Burghgrave, K. Burka, S. Burke, I. Burmeister, E. Busato, D. Büscher, V. Büscher, P. Bussey, J. M. Butler, A. I. Butt, C. M. Buttar, J. M. Butterworth, P. Butti, W. Buttinger, A. Buzatu, A. R. Buzykaev, S. Cabrera Urbán, D. Caforio, V. M. Cairo, O. Cakir, N. Calace, P. Calafiura, A. Calandri, G. Calderini, P. Calfayan, L. P. Caloba, D. Calvet, S. Calvet, T. P. Calvet, R. Camacho Toro, S. Camarda, P. Camarri, D. Cameron, R. Caminal Armadans, C. Camincher, S. Campana, M. Campanelli, A. Campoverde, V. Canale, A. Canepa, M. Cano Bret, J. Cantero, R. Cantrill, T. Cao, M. D. M. Capeans Garrido, I. Caprini, M. Caprini, M. Capua, R. Caputo, R. M. Carbone, R. Cardarelli, F. Cardillo, I. Carli, T. Carli, G. Carlino, L. Carminati, S. Caron, E. Carquin, G. D. Carrillo-Montoya, J. R. Carter, J. Carvalho, D. Casadei, M. P. Casado, M. Casolino, D. W. Casper, E. Castaneda-Miranda, A. Castelli, V. Castillo Gimenez, N. F. Castro, A. Catinaccio, J. R. Catmore, A. Cattai, J. Caudron, V. Cavaliere, D. Cavalli, M. Cavalli-Sforza, V. Cavasinni, F. Ceradini, L. Cerda Alberich, B. C. Cerio, A. S. Cerqueira, A. Cerri, L. Cerrito, F. Cerutti, M. Cerv, A. Cervelli, S. A. Cetin, A. Chafaq, D. Chakraborty, S. K. Chan, Y. L. Chan, P. Chang, J. D. Chapman, D. G. Charlton, A. Chatterjee, C. C. Chau, C. A. Chavez Barajas, S. Che, S. Cheatham, A. Chegwidden, S. Chekanov, S. V. Chekulaev, G. A. Chelkov, M. A. Chelstowska, C. Chen, H. Chen, K. Chen, S. Chen, S. Chen, X. Chen, Y. Chen, H. C. Cheng, H. J Cheng, Y. Cheng, A. Cheplakov, E. Cheremushkina, R. Cherkaoui El Moursli, V. Chernyatin, E. Cheu, L. Chevalier, V. Chiarella, G. Chiarelli, G. Chiodini, A. S. Chisholm, A. Chitan, M. V. Chizhov, K. Choi, A. R. Chomont, S. Chouridou, B. K. B. Chow, V. Christodoulou, D. Chromek-Burckhart, J. Chudoba, A. J. Chuinard, J. J. Chwastowski, L. Chytka, G. Ciapetti, A. K. Ciftci, D. Cinca, V. Cindro, I. A. Cioara, A. Ciocio, F. Cirotto, Z. H. Citron, M. Ciubancan, A. Clark, B. L. Clark, P. J. Clark, R. N. Clarke, C. Clement, Y. Coadou, M. Cobal, A. Coccaro, J. Cochran, L. Coffey, L. Colasurdo, B. Cole, S. Cole, A. P. Colijn, J. Collot, T. Colombo, G. Compostella, P. Conde Muiño, E. Coniavitis, S. H. Connell, I. A. Connelly, V. Consorti, S. Constantinescu, C. Conta, G. Conti, F. Conventi, M. Cooke, B. D. Cooper, A. M. Cooper-Sarkar, T. Cornelissen, M. Corradi, F. Corriveau, A. Corso-Radu, A. Cortes-Gonzalez, G. Cortiana, G. Costa, M. J. Costa, D. Costanzo, G. Cottin, G. Cowan, B. E. Cox, K. Cranmer, S. J. Crawley, G. Cree, S. Crépé-Renaudin, F. Crescioli, W. A. Cribbs, M. Crispin Ortuzar, M. Cristinziani, V. Croft, G. Crosetti, T. Cuhadar Donszelmann, J. Cummings, M. Curatolo, J. Cúth, C. Cuthbert, H. Czirr, P. Czodrowski, S. D’Auria, M. D’Onofrio, M. J. Da Cunha Sargedas De Sousa, C. Da Via, W. Dabrowski, T. Dai, O. Dale, F. Dallaire, C. Dallapiccola, M. Dam, J. R. Dandoy, N. P. Dang, A. C. Daniells, N. S. Dann, M. Danninger, M. Dano Hoffmann, V. Dao, G. Darbo, S. Darmora, J. Dassoulas, A. Dattagupta, W. Davey, C. David, T. Davidek, M. Davies, P. Davison, Y. Davygora, E. Dawe, I. Dawson, R. K. Daya-Ishmukhametova, K. De, R. de Asmundis, A. De Benedetti, S. De Castro, S. De Cecco, N. De Groot, P. de Jong, H. De la Torre, F. De Lorenzi, D. De Pedis, A. De Salvo, U. De Sanctis, A. De Santo, J. B. De Vivie De Regie, W. J. Dearnaley, R. Debbe, C. Debenedetti, D. V. Dedovich, I. Deigaard, J. Del Peso, T. Del Prete, D. Delgove, F. Deliot, C. M. Delitzsch, M. Deliyergiyev, A. Dell’Acqua, L. Dell’Asta, M. Dell’Orso, M. Della Pietra, D. della Volpe, M. Delmastro, P. A. Delsart, C. Deluca, D. A. DeMarco, S. Demers, M. Demichev, A. Demilly, S. P. Denisov, D. Denysiuk, D. Derendarz, J. E. Derkaoui, F. Derue, P. Dervan, K. Desch, C. Deterre, K. Dette, P. O. Deviveiros, A. Dewhurst, S. Dhaliwal, A. Di Ciaccio, L. Di Ciaccio, W. K. Di Clemente, C. Di Donato, A. Di Girolamo, B. Di Girolamo, B. Di Micco, R. Di Nardo, A. Di Simone, R. Di Sipio, D. Di Valentino, C. Diaconu, M. Diamond, F. A. Dias, M. A. Diaz, E. B. Diehl, J. Dietrich, S. Diglio, A. Dimitrievska, J. Dingfelder, P. Dita, S. Dita, F. Dittus, F. Djama, T. Djobava, J. I. Djuvsland, M. A. B. do Vale, D. Dobos, M. Dobre, C. Doglioni, T. Dohmae, J. Dolejsi, Z. Dolezal, B. A. Dolgoshein, M. Donadelli, S. Donati, P. Dondero, J. Donini, J. Dopke, A. Doria, M. T. Dova, A. T. Doyle, E. Drechsler, M. Dris, Y. Du, J. Duarte-Campderros, E. Duchovni, G. Duckeck, O. A. Ducu, D. Duda, A. Dudarev, L. Duflot, L. Duguid, M. Dührssen, M. Dunford, H. Duran Yildiz, M. Düren, A. Durglishvili, D. Duschinger, B. Dutta, M. Dyndal, C. Eckardt, K. M. Ecker, R. C. Edgar, W. Edson, N. C. Edwards, T. Eifert, G. Eigen, K. Einsweiler, T. Ekelof, M. El Kacimi, V. Ellajosyula, M. Ellert, S. Elles, F. Ellinghaus, A. A. Elliot, N. Ellis, J. Elmsheuser, M. Elsing, D. Emeliyanov, Y. Enari, O. C. Endner, M. Endo, J. S. Ennis, J. Erdmann, A. Ereditato, G. Ernis, J. Ernst, M. Ernst, S. Errede, E. Ertel, M. Escalier, H. Esch, C. Escobar, B. Esposito, A. I. Etienvre, E. Etzion, H. Evans, A. Ezhilov, F. Fabbri, L. Fabbri, G. Facini, R. M. Fakhrutdinov, S. Falciano, R. J. Falla, J. Faltova, Y. Fang, M. Fanti, A. Farbin, A. Farilla, C. Farina, T. Farooque, S. Farrell, S. M. Farrington, P. Farthouat, F. Fassi, P. Fassnacht, D. Fassouliotis, M. Faucci Giannelli, A. Favareto, W. J. Fawcett, L. Fayard, O. L. Fedin, W. Fedorko, S. Feigl, L. Feligioni, C. Feng, E. J. Feng, H. Feng, A. B. Fenyuk, L. Feremenga, P. Fernandez Martinez, S. Fernandez Perez, J. Ferrando, A. Ferrari, P. Ferrari, R. Ferrari, D. E. Ferreira de Lima, A. Ferrer, D. Ferrere, C. Ferretti, A. Ferretto Parodi, F. Fiedler, A. Filipčič, M. Filipuzzi, F. Filthaut, M. Fincke-Keeler, K. D. Finelli, M. C. N. Fiolhais, L. Fiorini, A. Firan, A. Fischer, C. Fischer, J. Fischer, W. C. Fisher, N. Flaschel, I. Fleck, P. Fleischmann, G. T. Fletcher, G. Fletcher, R. R. M. Fletcher, T. Flick, A. Floderus, L. R. Flores Castillo, M. J. Flowerdew, G. T. Forcolin, A. Formica, A. Forti, A. G. Foster, D. Fournier, H. Fox, S. Fracchia, P. Francavilla, M. Franchini, D. Francis, L. Franconi, M. Franklin, M. Frate, M. Fraternali, D. Freeborn, S. M. Fressard-Batraneanu, F. Friedrich, D. Froidevaux, J. A. Frost, C. Fukunaga, E. Fullana Torregrosa, T. Fusayasu, J. Fuster, C. Gabaldon, O. Gabizon, A. Gabrielli, A. Gabrielli, G. P. Gach, S. Gadatsch, S. Gadomski, G. Gagliardi, L. G. Gagnon, P. Gagnon, C. Galea, B. Galhardo, E. J. Gallas, B. J. Gallop, P. Gallus, G. Galster, K. K. Gan, J. Gao, Y. Gao, Y. S. Gao, F. M. Garay Walls, C. García, J. E. García Navarro, M. Garcia-Sciveres, R. W. Gardner, N. Garelli, V. Garonne, A. Gascon Bravo, C. Gatti, A. Gaudiello, G. Gaudio, B. Gaur, L. Gauthier, I. L. Gavrilenko, C. Gay, G. Gaycken, E. N. Gazis, Z. Gecse, C. N. P. Gee, Ch. Geich-Gimbel, M. P. Geisler, C. Gemme, M. H. Genest, C. Geng, S. Gentile, S. George, D. Gerbaudo, A. Gershon, S. Ghasemi, H. Ghazlane, B. Giacobbe, S. Giagu, P. Giannetti, B. Gibbard, S. M. Gibson, M. Gignac, M. Gilchriese, T. P. S. Gillam, D. Gillberg, G. Gilles, D. M. Gingrich, N. Giokaris, M. P. Giordani, F. M. Giorgi, F. M. Giorgi, P. F. Giraud, P. Giromini, D. Giugni, C. Giuliani, M. Giulini, B. K. Gjelsten, S. Gkaitatzis, I. Gkialas, E. L. Gkougkousis, L. K. Gladilin, C. Glasman, J. Glatzer, P. C. F. Glaysher, A. Glazov, M. Goblirsch-Kolb, J. Godlewski, S. Goldfarb, T. Golling, D. Golubkov, A. Gomes, R. Gonçalo, J. Goncalves Pinto Firmino Da Costa, L. Gonella, A. Gongadze, S. González de la Hoz, G. Gonzalez Parra, S. Gonzalez-Sevilla, L. Goossens, P. A. Gorbounov, H. A. Gordon, I. Gorelov, B. Gorini, E. Gorini, A. Gorišek, E. Gornicki, A. T. Goshaw, C. Gössling, M. I. Gostkin, C. R. Goudet, D. Goujdami, A. G. Goussiou, N. Govender, E. Gozani, L. Graber, I. Grabowska-Bold, P. O. J. Gradin, P. Grafström, J. Gramling, E. Gramstad, S. Grancagnolo, V. Gratchev, H. M. Gray, E. Graziani, Z. D. Greenwood, C. Grefe, K. Gregersen, I. M. Gregor, P. Grenier, K. Grevtsov, J. Griffiths, A. A. Grillo, K. Grimm, S. Grinstein, Ph. Gris, J. -F. Grivaz, S. Groh, J. P. Grohs, E. Gross, J. Grosse-Knetter, G. C. Grossi, Z. J. Grout, L. Guan, W. Guan, J. Guenther, F. Guescini, D. Guest, O. Gueta, E. Guido, T. Guillemin, S. Guindon, U. Gul, C. Gumpert, J. Guo, Y. Guo, S. Gupta, G. Gustavino, P. Gutierrez, N. G. Gutierrez Ortiz, C. Gutschow, C. Guyot, C. Gwenlan, C. B. Gwilliam, A. Haas, C. Haber, H. K. Hadavand, N. Haddad, A. Hadef, P. Haefner, S. Hageböck, Z. Hajduk, H. Hakobyan, M. Haleem, J. Haley, D. Hall, G. Halladjian, G. D. Hallewell, K. Hamacher, P. Hamal, K. Hamano, A. Hamilton, G. N. Hamity, P. G. Hamnett, L. Han, K. Hanagaki, K. Hanawa, M. Hance, B. Haney, P. Hanke, R. Hanna, J. B. Hansen, J. D. Hansen, M. C. Hansen, P. H. Hansen, K. Hara, A. S. Hard, T. Harenberg, F. Hariri, S. Harkusha, R. D. Harrington, P. F. Harrison, F. Hartjes, N. M. Hartmann, M. Hasegawa, Y. Hasegawa, A. Hasib, S. Hassani, S. Haug, R. Hauser, L. Hauswald, M. Havranek, C. M. Hawkes, R. J. Hawkings, A. D. Hawkins, D. Hayden, C. P. Hays, J. M. Hays, H. S. Hayward, S. J. Haywood, S. J. Head, T. Heck, V. Hedberg, L. Heelan, S. Heim, T. Heim, B. Heinemann, J. J. Heinrich, L. Heinrich, C. Heinz, J. Hejbal, L. Helary, S. Hellman, C. Helsens, J. Henderson, R. C. W. Henderson, Y. Heng, S. Henkelmann, A. M. Henriques Correia, S. Henrot-Versille, G. H. Herbert, Y. Hernández Jiménez, G. Herten, R. Hertenberger, L. Hervas, G. G. Hesketh, N. P. Hessey, J. W. Hetherly, R. Hickling, E. Higón-Rodriguez, E. Hill, J. C. Hill, K. H. Hiller, S. J. Hillier, I. Hinchliffe, E. Hines, R. R. Hinman, M. Hirose, D. Hirschbuehl, J. Hobbs, N. Hod, M. C. Hodgkinson, P. Hodgson, A. Hoecker, M. R. Hoeferkamp, F. Hoenig, M. Hohlfeld, D. Hohn, T. R. Holmes, M. Homann, T. M. Hong, B. H. Hooberman, W. H. Hopkins, Y. Horii, A. J. Horton, J-Y. Hostachy, S. Hou, A. Hoummada, J. Howard, J. Howarth, M. Hrabovsky, I. Hristova, J. Hrivnac, T. Hryn’ova, A. Hrynevich, C. Hsu, P. J. Hsu, S. -C. Hsu, D. Hu, Q. Hu, Y. Huang, Z. Hubacek, F. Hubaut, F. Huegging, T. B. Huffman, E. W. Hughes, G. Hughes, M. Huhtinen, T. A. Hülsing, N. Huseynov, J. Huston, J. Huth, G. Iacobucci, G. Iakovidis, I. Ibragimov, L. Iconomidou-Fayard, E. Ideal, Z. Idrissi, P. Iengo, O. Igonkina, T. Iizawa, Y. Ikegami, M. Ikeno, Y. Ilchenko, D. Iliadis, N. Ilic, T. Ince, G. Introzzi, P. Ioannou, M. Iodice, K. Iordanidou, V. Ippolito, A. Irles Quiles, C. Isaksson, M. Ishino, M. Ishitsuka, R. Ishmukhametov, C. Issever, S. Istin, F. Ito, J. M. Iturbe Ponce, R. Iuppa, J. Ivarsson, W. Iwanski, H. Iwasaki, J. M. Izen, V. Izzo, S. Jabbar, B. Jackson, M. Jackson, P. Jackson, V. Jain, K. B. Jakobi, K. Jakobs, S. Jakobsen, T. Jakoubek, D. O. Jamin, D. K. Jana, E. Jansen, R. Jansky, J. Janssen, M. Janus, G. Jarlskog, N. Javadov, T. Javůrek, F. Jeanneau, L. Jeanty, J. Jejelava, G. -Y. Jeng, D. Jennens, P. Jenni, J. Jentzsch, C. Jeske, S. Jézéquel, H. Ji, J. Jia, H. Jiang, Y. Jiang, S. Jiggins, J. Jimenez Pena, S. Jin, A. Jinaru, O. Jinnouchi, P. Johansson, K. A. Johns, W. J. Johnson, K. Jon-And, G. Jones, R. W. L. Jones, S. Jones, T. J. Jones, J. Jongmanns, P. M. Jorge, J. Jovicevic, X. Ju, A. Juste Rozas, M. K. Köhler, A. Kaczmarska, M. Kado, H. Kagan, M. Kagan, S. J. Kahn, E. Kajomovitz, C. W. Kalderon, A. Kaluza, S. Kama, A. Kamenshchikov, N. Kanaya, S. Kaneti, V. A. Kantserov, J. Kanzaki, B. Kaplan, L. S. Kaplan, A. Kapliy, D. Kar, K. Karakostas, A. Karamaoun, N. Karastathis, M. J. Kareem, E. Karentzos, M. Karnevskiy, S. N. Karpov, Z. M. Karpova, K. Karthik, V. Kartvelishvili, A. N. Karyukhin, K. Kasahara, L. Kashif, R. D. Kass, A. Kastanas, Y. Kataoka, C. Kato, A. Katre, J. Katzy, K. Kawagoe, T. Kawamoto, G. Kawamura, S. Kazama, V. F. Kazanin, R. Keeler, R. Kehoe, J. S. Keller, J. J. Kempster, K Kentaro, H. Keoshkerian, O. Kepka, B. P. Kerševan, S. Kersten, R. A. Keyes, F. Khalil-zada, H. Khandanyan, A. Khanov, A. G. Kharlamov, T. J. Khoo, V. Khovanskiy, E. Khramov, J. Khubua, S. Kido, H. Y. Kim, S. H. Kim, Y. K. Kim, N. Kimura, O. M. Kind, B. T. King, M. King, S. B. King, J. Kirk, A. E. Kiryunin, T. Kishimoto, D. Kisielewska, F. Kiss, K. Kiuchi, O. Kivernyk, E. Kladiva, M. H. Klein, M. Klein, U. Klein, K. Kleinknecht, P. Klimek, A. Klimentov, R. Klingenberg, J. A. Klinger, T. Klioutchnikova, E. -E. Kluge, P. Kluit, S. Kluth, J. Knapik, E. Kneringer, E. B. F. G. Knoops, A. Knue, A. Kobayashi, D. Kobayashi, T. Kobayashi, M. Kobel, M. Kocian, P. Kodys, T. Koffas, E. Koffeman, L. A. Kogan, T. Koi, H. Kolanoski, M. Kolb, I. Koletsou, A. A. Komar, Y. Komori, T. Kondo, N. Kondrashova, K. Köneke, A. C. König, T. Kono, R. Konoplich, N. Konstantinidis, R. Kopeliansky, S. Koperny, L. Köpke, A. K. Kopp, K. Korcyl, K. Kordas, A. Korn, A. A. Korol, I. Korolkov, E. V. Korolkova, O. Kortner, S. Kortner, T. Kosek, V. V. Kostyukhin, V. M. Kotov, A. Kotwal, A. Kourkoumeli-Charalampidi, C. Kourkoumelis, V. Kouskoura, A. Koutsman, A. B. Kowalewska, R. Kowalewski, T. Z. Kowalski, W. Kozanecki, A. S. Kozhin, V. A. Kramarenko, G. Kramberger, D. Krasnopevtsev, M. W. Krasny, A. Krasznahorkay, J. K. Kraus, A. Kravchenko, M. Kretz, J. Kretzschmar, K. Kreutzfeldt, P. Krieger, K. Krizka, K. Kroeninger, H. Kroha, J. Kroll, J. Kroseberg, J. Krstic, U. Kruchonak, H. Krüger, N. Krumnack, A. Kruse, M. C. Kruse, M. Kruskal, T. Kubota, H. Kucuk, S. Kuday, J. T. Kuechler, S. Kuehn, A. Kugel, F. Kuger, A. Kuhl, T. Kuhl, V. Kukhtin, R. Kukla, Y. Kulchitsky, S. Kuleshov, M. Kuna, T. Kunigo, A. Kupco, H. Kurashige, Y. A. Kurochkin, V. Kus, E. S. Kuwertz, M. Kuze, J. Kvita, T. Kwan, D. Kyriazopoulos, A. La Rosa, J. L. La Rosa Navarro, L. La Rotonda, C. Lacasta, F. Lacava, J. Lacey, H. Lacker, D. Lacour, V. R. Lacuesta, E. Ladygin, R. Lafaye, B. Laforge, T. Lagouri, S. Lai, S. Lammers, W. Lampl, E. Lançon, U. Landgraf, M. P. J. Landon, V. S. Lang, J. C. Lange, A. J. Lankford, F. Lanni, K. Lantzsch, A. Lanza, S. Laplace, C. Lapoire, J. F. Laporte, T. Lari, F. Lasagni Manghi, M. Lassnig, P. Laurelli, W. Lavrijsen, A. T. Law, P. Laycock, T. Lazovich, M. Lazzaroni, O. Le Dortz, E. Le Guirriec, E. Le Menedeu, E. P. Le Quilleuc, M. LeBlanc, T. LeCompte, F. Ledroit-Guillon, C. A. Lee, S. C. Lee, L. Lee, G. Lefebvre, M. Lefebvre, F. Legger, C. Leggett, A. Lehan, G. Lehmann Miotto, X. Lei, W. A. Leight, A. Leisos, A. G. Leister, M. A. L. Leite, R. Leitner, D. Lellouch, B. Lemmer, K. J. C. Leney, T. Lenz, B. Lenzi, R. Leone, S. Leone, C. Leonidopoulos, S. Leontsinis, G. Lerner, C. Leroy, A. A. J. Lesage, C. G. Lester, M. Levchenko, J. Levêque, D. Levin, L. J. Levinson, M. Levy, A. M. Leyko, M. Leyton, B. Li, H. Li, H. L. Li, L. Li, L. Li, Q. Li, S. Li, X. Li, Y. Li, Z. Liang, H. Liao, B. Liberti, A. Liblong, P. Lichard, K. Lie, J. Liebal, W. Liebig, C. Limbach, A. Limosani, S. C. Lin, T. H. Lin, B. E. Lindquist, E. Lipeles, A. Lipniacka, M. Lisovyi, T. M. Liss, D. Lissauer, A. Lister, A. M. Litke, B. Liu, D. Liu, H. Liu, H. Liu, J. Liu, J. B. Liu, K. Liu, L. Liu, M. Liu, M. Liu, Y. L. Liu, Y. Liu, M. Livan, A. Lleres, J. Llorente Merino, S. L. Lloyd, F. Lo Sterzo, E. Lobodzinska, P. Loch, W. S. Lockman, F. K. Loebinger, A. E. Loevschall-Jensen, K. M. Loew, A. Loginov, T. Lohse, K. Lohwasser, M. Lokajicek, B. A. Long, J. D. Long, R. E. Long, L. Longo, K. A. Looper, L. Lopes, D. Lopez Mateos, B. Lopez Paredes, I. Lopez Paz, A. Lopez Solis, J. Lorenz, N. Lorenzo Martinez, M. Losada, P. J. Lösel, X. Lou, A. Lounis, J. Love, P. A. Love, H. Lu, N. Lu, H. J. Lubatti, C. Luci, A. Lucotte, C. Luedtke, F. Luehring, W. Lukas, L. Luminari, O. Lundberg, B. Lund-Jensen, D. Lynn, R. Lysak, E. Lytken, V. Lyubushkin, H. Ma, L. L. Ma, G. Maccarrone, A. Macchiolo, C. M. Macdonald, B. Maček, J. Machado Miguens, D. Madaffari, R. Madar, H. J. Maddocks, W. F. Mader, A. Madsen, J. Maeda, S. Maeland, T. Maeno, A. Maevskiy, E. Magradze, J. Mahlstedt, C. Maiani, C. Maidantchik, A. A. Maier, T. Maier, A. Maio, S. Majewski, Y. Makida, N. Makovec, B. Malaescu, Pa. Malecki, V. P. Maleev, F. Malek, U. Mallik, D. Malon, C. Malone, S. Maltezos, S. Malyukov, J. Mamuzic, G. Mancini, B. Mandelli, L. Mandelli, I. Mandić, J. Maneira, L. Manhaes de Andrade Filho, J. Manjarres Ramos, A. Mann, B. Mansoulie, R. Mantifel, M. Mantoani, S. Manzoni, L. Mapelli, G. Marceca, L. March, G. Marchiori, M. Marcisovsky, M. Marjanovic, D. E. Marley, F. Marroquim, S. P. Marsden, Z. Marshall, L. F. Marti, S. Marti-Garcia, B. Martin, T. A. Martin, V. J. Martin, B. Martin dit Latour, M. Martinez, S. Martin-Haugh, V. S. Martoiu, A. C. Martyniuk, M. Marx, F. Marzano, A. Marzin, L. Masetti, T. Mashimo, R. Mashinistov, J. Masik, A. L. Maslennikov, I. Massa, L. Massa, P. Mastrandrea, A. Mastroberardino, T. Masubuchi, P. Mättig, J. Mattmann, J. Maurer, S. J. Maxfield, D. A. Maximov, R. Mazini, S. M. Mazza, N. C. Mc Fadden, G. Mc Goldrick, S. P. Mc Kee, A. McCarn, R. L. McCarthy, T. G. McCarthy, L. I. McClymont, K. W. McFarlane, J. A. Mcfayden, G. Mchedlidze, S. J. McMahon, R. A. McPherson, M. Medinnis, S. Meehan, S. Mehlhase, A. Mehta, K. Meier, C. Meineck, B. Meirose, B. R. Mellado Garcia, F. Meloni, A. Mengarelli, S. Menke, E. Meoni, K. M. Mercurio, S. Mergelmeyer, P. Mermod, L. Merola, C. Meroni, F. S. Merritt, A. Messina, J. Metcalfe, A. S. Mete, C. Meyer, C. Meyer, J-P. Meyer, J. Meyer, H. Meyer Zu Theenhausen, R. P. Middleton, S. Miglioranzi, L. Mijović, G. Mikenberg, M. Mikestikova, M. Mikuž, M. Milesi, A. Milic, D. W. Miller, C. Mills, A. Milov, D. A. Milstead, A. A. Minaenko, Y. Minami, I. A. Minashvili, A. I. Mincer, B. Mindur, M. Mineev, Y. Ming, L. M. Mir, K. P. Mistry, T. Mitani, J. Mitrevski, V. A. Mitsou, A. Miucci, P. S. Miyagawa, J. U. Mjörnmark, T. Moa, K. Mochizuki, S. Mohapatra, W. Mohr, S. Molander, R. Moles-Valls, R. Monden, M. C. Mondragon, K. Mönig, J. Monk, E. Monnier, A. Montalbano, J. Montejo Berlingen, F. Monticelli, S. Monzani, R. W. Moore, N. Morange, D. Moreno, M. Moreno Llácer, P. Morettini, D. Mori, T. Mori, M. Morii, M. Morinaga, V. Morisbak, S. Moritz, A. K. Morley, G. Mornacchi, J. D. Morris, S. S. Mortensen, L. Morvaj, M. Mosidze, J. Moss, K. Motohashi, R. Mount, E. Mountricha, S. V. Mouraviev, E. J. W. Moyse, S. Muanza, R. D. Mudd, F. Mueller, J. Mueller, R. S. P. Mueller, T. Mueller, D. Muenstermann, P. Mullen, G. A. Mullier, F. J. Munoz Sanchez, J. A. Murillo Quijada, W. J. Murray, H. Musheghyan, A. G. Myagkov, M. Myska, B. P. Nachman, O. Nackenhorst, J. Nadal, K. Nagai, R. Nagai, Y. Nagai, K. Nagano, Y. Nagasaka, K. Nagata, M. Nagel, E. Nagy, A. M. Nairz, Y. Nakahama, K. Nakamura, T. Nakamura, I. Nakano, H. Namasivayam, R. F. Naranjo Garcia, R. Narayan, D. I. Narrias Villar, I. Naryshkin, T. Naumann, G. Navarro, R. Nayyar, H. A. Neal, P. Yu. Nechaeva, T. J. Neep, P. D. Nef, A. Negri, M. Negrini, S. Nektarijevic, C. Nellist, A. Nelson, S. Nemecek, P. Nemethy, A. A. Nepomuceno, M. Nessi, M. S. Neubauer, M. Neumann, R. M. Neves, P. Nevski, P. R. Newman, D. H. Nguyen, R. B. Nickerson, R. Nicolaidou, B. Nicquevert, J. Nielsen, A. Nikiforov, V. Nikolaenko, I. Nikolic-Audit, K. Nikolopoulos, J. K. Nilsen, P. Nilsson, Y. Ninomiya, A. Nisati, R. Nisius, T. Nobe, L. Nodulman, M. Nomachi, I. Nomidis, T. Nooney, S. Norberg, M. Nordberg, N. Norjoharuddeen, O. Novgorodova, S. Nowak, M. Nozaki, L. Nozka, K. Ntekas, E. Nurse, F. Nuti, F. O’grady, D. C. O’Neil, A. A. O’Rourke, V. O’Shea, F. G. Oakham, H. Oberlack, T. Obermann, J. Ocariz, A. Ochi, I. Ochoa, J. P. Ochoa-Ricoux, S. Oda, S. Odaka, H. Ogren, A. Oh, S. H. Oh, C. C. Ohm, H. Ohman, H. Oide, H. Okawa, Y. Okumura, T. Okuyama, A. Olariu, L. F. Oleiro Seabra, S. A. Olivares Pino, D. Oliveira Damazio, A. Olszewski, J. Olszowska, A. Onofre, K. Onogi, P. U. E. Onyisi, C. J. Oram, M. J. Oreglia, Y. Oren, D. Orestano, N. Orlando, R. S. Orr, B. Osculati, R. Ospanov, G. Otero y Garzon, H. Otono, M. Ouchrif, F. Ould-Saada, A. Ouraou, K. P. Oussoren, Q. Ouyang, A. Ovcharova, M. Owen, R. E. Owen, V. E. Ozcan, N. Ozturk, K. Pachal, A. Pacheco Pages, C. Padilla Aranda, M. Pagáčová, S. Pagan Griso, F. Paige, P. Pais, K. Pajchel, G. Palacino, S. Palestini, M. Palka, D. Pallin, A. Palma, E. St. Panagiotopoulou, C. E. Pandini, J. G. Panduro Vazquez, P. Pani, S. Panitkin, D. Pantea, L. Paolozzi, Th. D. Papadopoulou, K. Papageorgiou, A. Paramonov, D. Paredes Hernandez, M. A. Parker, K. A. Parker, F. Parodi, J. A. Parsons, U. Parzefall, V. R. Pascuzzi, E. Pasqualucci, S. Passaggio, F. Pastore, Fr. Pastore, G. Pásztor, S. Pataraia, N. D. Patel, J. R. Pater, T. Pauly, J. Pearce, B. Pearson, L. E. Pedersen, M. Pedersen, S. Pedraza Lopez, R. Pedro, S. V. Peleganchuk, D. Pelikan, O. Penc, C. Peng, H. Peng, J. Penwell, B. S. Peralva, M. M. Perego, D. V. Perepelitsa, E. Perez Codina, L. Perini, H. Pernegger, S. Perrella, R. Peschke, V. D. Peshekhonov, K. Peters, R. F. Y. Peters, B. A. Petersen, T. C. Petersen, E. Petit, A. Petridis, C. Petridou, P. Petroff, E. Petrolo, M. Petrov, F. Petrucci, N. E. Pettersson, A. Peyaud, R. Pezoa, P. W. Phillips, G. Piacquadio, E. Pianori, A. Picazio, E. Piccaro, M. Piccinini, M. A. Pickering, R. Piegaia, J. E. Pilcher, A. D. Pilkington, A. W. J. Pin, J. Pina, M. Pinamonti, J. L. Pinfold, A. Pingel, S. Pires, H. Pirumov, M. Pitt, L. Plazak, M. -A. Pleier, V. Pleskot, E. Plotnikova, P. Plucinski, D. Pluth, R. Poettgen, L. Poggioli, D. Pohl, G. Polesello, A. Poley, A. Policicchio, R. Polifka, A. Polini, C. S. Pollard, V. Polychronakos, K. Pommès, L. Pontecorvo, B. G. Pope, G. A. Popeneciu, D. S. Popovic, A. Poppleton, S. Pospisil, K. Potamianos, I. N. Potrap, C. J. Potter, C. T. Potter, G. Poulard, J. Poveda, V. Pozdnyakov, M. E. Pozo Astigarraga, P. Pralavorio, A. Pranko, S. Prell, D. Price, L. E. Price, M. Primavera, S. Prince, M. Proissl, K. Prokofiev, F. Prokoshin, S. Protopopescu, J. Proudfoot, M. Przybycien, D. Puddu, D. Puldon, M. Purohit, P. Puzo, J. Qian, G. Qin, Y. Qin, A. Quadt, W. B. Quayle, M. Queitsch-Maitland, D. Quilty, S. Raddum, V. Radeka, V. Radescu, S. K. Radhakrishnan, P. Radloff, P. Rados, F. Ragusa, G. Rahal, S. Rajagopalan, M. Rammensee, C. Rangel-Smith, M. G. Ratti, F. Rauscher, S. Rave, T. Ravenscroft, M. Raymond, A. L. Read, N. P. Readioff, D. M. Rebuzzi, A. Redelbach, G. Redlinger, R. Reece, K. Reeves, L. Rehnisch, J. Reichert, H. Reisin, C. Rembser, H. Ren, M. Rescigno, S. Resconi, O. L. Rezanova, P. Reznicek, R. Rezvani, R. Richter, S. Richter, E. Richter-Was, O. Ricken, M. Ridel, P. Rieck, C. J. Riegel, J. Rieger, O. Rifki, M. Rijssenbeek, A. Rimoldi, L. Rinaldi, B. Ristić, E. Ritsch, I. Riu, F. Rizatdinova, E. Rizvi, C. Rizzi, S. H. Robertson, A. Robichaud-Veronneau, D. Robinson, J. E. M. Robinson, A. Robson, C. Roda, Y. Rodina, A. Rodriguez Perez, D. Rodriguez Rodriguez, S. Roe, C. S. Rogan, O. Røhne, A. Romaniouk, M. Romano, S. M. Romano Saez, E. Romero Adam, N. Rompotis, M. Ronzani, L. Roos, E. Ros, S. Rosati, K. Rosbach, P. Rose, O. Rosenthal, V. Rossetti, E. Rossi, L. P. Rossi, J. H. N. Rosten, R. Rosten, M. Rotaru, I. Roth, J. Rothberg, D. Rousseau, C. R. Royon, A. Rozanov, Y. Rozen, X. Ruan, F. Rubbo, I. Rubinskiy, V. I. Rud, M. S. Rudolph, F. Rühr, A. Ruiz-Martinez, Z. Rurikova, N. A. Rusakovich, A. Ruschke, H. L. Russell, J. P. Rutherfoord, N. Ruthmann, Y. F. Ryabov, M. Rybar, G. Rybkin, S. Ryu, A. Ryzhov, A. F. Saavedra, G. Sabato, S. Sacerdoti, H. F-W. Sadrozinski, R. Sadykov, F. Safai Tehrani, P. Saha, M. Sahinsoy, M. Saimpert, T. Saito, H. Sakamoto, Y. Sakurai, G. Salamanna, A. Salamon, J. E. Salazar Loyola, D. Salek, P. H. Sales De Bruin, D. Salihagic, A. Salnikov, J. Salt, D. Salvatore, F. Salvatore, A. Salvucci, A. Salzburger, D. Sammel, D. Sampsonidis, A. Sanchez, J. Sánchez, V. Sanchez Martinez, H. Sandaker, R. L. Sandbach, H. G. Sander, M. P. Sanders, M. Sandhoff, C. Sandoval, R. Sandstroem, D. P. C. Sankey, M. Sannino, A. Sansoni, C. Santoni, R. Santonico, H. Santos, I. Santoyo Castillo, K. Sapp, A. Sapronov, J. G. Saraiva, B. Sarrazin, O. Sasaki, Y. Sasaki, K. Sato, G. Sauvage, E. Sauvan, G. Savage, P. Savard, C. Sawyer, L. Sawyer, J. Saxon, C. Sbarra, A. Sbrizzi, T. Scanlon, D. A. Scannicchio, M. Scarcella, V. Scarfone, J. Schaarschmidt, P. Schacht, D. Schaefer, R. Schaefer, J. Schaeffer, S. Schaepe, S. Schaetzel, U. Schäfer, A. C. Schaffer, D. Schaile, R. D. Schamberger, V. Scharf, V. A. Schegelsky, D. Scheirich, M. Schernau, C. Schiavi, C. Schillo, M. Schioppa, S. Schlenker, K. Schmieden, C. Schmitt, S. Schmitt, S. Schmitz, B. Schneider, Y. J. Schnellbach, U. Schnoor, L. Schoeffel, A. Schoening, B. D. Schoenrock, E. Schopf, A. L. S. Schorlemmer, M. Schott, D. Schouten, J. Schovancova, S. Schramm, M. Schreyer, N. Schuh, M. J. Schultens, H. -C. Schultz-Coulon, H. Schulz, M. Schumacher, B. A. Schumm, Ph. Schune, C. Schwanenberger, A. Schwartzman, T. A. Schwarz, Ph. Schwegler, H. Schweiger, Ph. Schwemling, R. Schwienhorst, J. Schwindling, T. Schwindt, G. Sciolla, F. Scuri, F. Scutti, J. Searcy, P. Seema, S. C. Seidel, A. Seiden, F. Seifert, J. M. Seixas, G. Sekhniaidze, K. Sekhon, S. J. Sekula, D. M. Seliverstov, N. Semprini-Cesari, C. Serfon, L. Serin, L. Serkin, M. Sessa, R. Seuster, H. Severini, T. Sfiligoj, F. Sforza, A. Sfyrla, E. Shabalina, N. W. Shaikh, L. Y. Shan, R. Shang, J. T. Shank, M. Shapiro, P. B. Shatalov, K. Shaw, S. M. Shaw, A. Shcherbakova, C. Y. Shehu, P. Sherwood, L. Shi, S. Shimizu, C. O. Shimmin, M. Shimojima, M. Shiyakova, A. Shmeleva, D. Shoaleh Saadi, M. J. Shochet, S. Shojaii, S. Shrestha, E. Shulga, M. A. Shupe, P. Sicho, P. E. Sidebo, O. Sidiropoulou, D. Sidorov, A. Sidoti, F. Siegert, Dj. Sijacki, J. Silva, S. B. Silverstein, V. Simak, O. Simard, Lj. Simic, S. Simion, E. Simioni, B. Simmons, D. Simon, M. Simon, P. Sinervo, N. B. Sinev, M. Sioli, G. Siragusa, S. Yu. Sivoklokov, J. Sjölin, T. B. Sjursen, M. B. Skinner, H. P. Skottowe, P. Skubic, M. Slater, T. Slavicek, M. Slawinska, K. Sliwa, R. Slovak, V. Smakhtin, B. H. Smart, L. Smestad, S. Yu. Smirnov, Y. Smirnov, L. N. Smirnova, O. Smirnova, M. N. K. Smith, R. W. Smith, M. Smizanska, K. Smolek, A. A. Snesarev, G. Snidero, S. Snyder, R. Sobie, F. Socher, A. Soffer, D. A. Soh, G. Sokhrannyi, C. A. Solans Sanchez, M. Solar, E. Yu. Soldatov, U. Soldevila, A. A. Solodkov, A. Soloshenko, O. V. Solovyanov, V. Solovyev, P. Sommer, H. Son, H. Y. Song, A. Sood, A. Sopczak, V. Sopko, V. Sorin, D. Sosa, C. L. Sotiropoulou, R. Soualah, A. M. Soukharev, D. South, B. C. Sowden, S. Spagnolo, M. Spalla, M. Spangenberg, F. Spanò, D. Sperlich, F. Spettel, R. Spighi, G. Spigo, L. A. Spiller, M. Spousta, R. D. St. Denis, A. Stabile, J. Stahlman, R. Stamen, S. Stamm, E. Stanecka, R. W. Stanek, C. Stanescu, M. Stanescu-Bellu, M. M. Stanitzki, S. Stapnes, E. A. Starchenko, G. H. Stark, J. Stark, P. Staroba, P. Starovoitov, S. Stärz, R. Staszewski, P. Steinberg, B. Stelzer, H. J. Stelzer, O. Stelzer-Chilton, H. Stenzel, G. A. Stewart, J. A. Stillings, M. C. Stockton, M. Stoebe, G. Stoicea, P. Stolte, S. Stonjek, A. R. Stradling, A. Straessner, M. E. Stramaglia, J. Strandberg, S. Strandberg, A. Strandlie, M. Strauss, P. Strizenec, R. Ströhmer, D. M. Strom, R. Stroynowski, A. Strubig, S. A. Stucci, B. Stugu, N. A. Styles, D. Su, J. Su, R. Subramaniam, S. Suchek, Y. Sugaya, M. Suk, V. V. Sulin, S. Sultansoy, T. Sumida, S. Sun, X. Sun, J. E. Sundermann, K. Suruliz, G. Susinno, M. R. Sutton, S. Suzuki, M. Svatos, M. Swiatlowski, I. Sykora, T. Sykora, D. Ta, C. Taccini, K. Tackmann, J. Taenzer, A. Taffard, R. Tafirout, N. Taiblum, H. Takai, R. Takashima, H. Takeda, T. Takeshita, Y. Takubo, M. Talby, A. A. Talyshev, J. Y. C. Tam, K. G. Tan, J. Tanaka, R. Tanaka, S. Tanaka, B. B. Tannenwald, S. Tapia Araya, S. Tapprogge, S. Tarem, G. F. Tartarelli, P. Tas, M. Tasevsky, T. Tashiro, E. Tassi, A. Tavares Delgado, Y. Tayalati, A. C. Taylor, G. N. Taylor, P. T. E. Taylor, W. Taylor, F. A. Teischinger, P. Teixeira-Dias, K. K. Temming, D. Temple, H. Ten Kate, P. K. Teng, J. J. Teoh, F. Tepel, S. Terada, K. Terashi, J. Terron, S. Terzo, M. Testa, R. J. Teuscher, T. Theveneaux-Pelzer, J. P. Thomas, J. Thomas-Wilsker, E. N. Thompson, P. D. Thompson, R. J. Thompson, A. S. Thompson, L. A. Thomsen, E. Thomson, M. Thomson, M. J. Tibbetts, R. E. Ticse Torres, V. O. Tikhomirov, Yu. A. Tikhonov, S. Timoshenko, P. Tipton, S. Tisserant, K. Todome, T. Todorov, S. Todorova-Nova, J. Tojo, S. Tokár, K. Tokushuku, E. Tolley, L. Tomlinson, M. Tomoto, L. Tompkins, K. Toms, B. Tong, E. Torrence, H. Torres, E. Torró Pastor, J. Toth, F. Touchard, D. R. Tovey, T. Trefzger, A. Tricoli, I. M. Trigger, S. Trincaz-Duvoid, M. F. Tripiana, W. Trischuk, B. Trocmé, A. Trofymov, C. Troncon, M. Trottier-McDonald, M. Trovatelli, L. Truong, M. Trzebinski, A. Trzupek, J. C-L. Tseng, P. V. Tsiareshka, G. Tsipolitis, N. Tsirintanis, S. Tsiskaridze, V. Tsiskaridze, E. G. Tskhadadze, K. M. Tsui, I. I. Tsukerman, V. Tsulaia, S. Tsuno, D. Tsybychev, A. Tudorache, V. Tudorache, A. N. Tuna, S. A. Tupputi, S. Turchikhin, D. Turecek, D. Turgeman, R. Turra, A. J. Turvey, P. M. Tuts, M. Tylmad, M. Tyndel, G. Ucchielli, I. Ueda, R. Ueno, M. Ughetto, F. Ukegawa, G. Unal, A. Undrus, G. Unel, F. C. Ungaro, Y. Unno, C. Unverdorben, J. Urban, P. Urquijo, P. Urrejola, G. Usai, A. Usanova, L. Vacavant, V. Vacek, B. Vachon, C. Valderanis, E. Valdes Santurio, N. Valencic, S. Valentinetti, A. Valero, L. Valery, S. Valkar, S. Vallecorsa, J. A. Valls Ferrer, W. Van Den Wollenberg, P. C. Van Der Deijl, R. van der Geer, H. van der Graaf, N. van Eldik, P. van Gemmeren, J. Van Nieuwkoop, I. van Vulpen, M. C. van Woerden, M. Vanadia, W. Vandelli, R. Vanguri, A. Vaniachine, P. Vankov, G. Vardanyan, R. Vari, E. W. Varnes, T. Varol, D. Varouchas, A. Vartapetian, K. E. Varvell, F. Vazeille, T. Vazquez Schroeder, J. Veatch, L. M. Veloce, F. Veloso, S. Veneziano, A. Ventura, M. Venturi, N. Venturi, A. Venturini, V. Vercesi, M. Verducci, W. Verkerke, J. C. Vermeulen, A. Vest, M. C. Vetterli, O. Viazlo, I. Vichou, T. Vickey, O. E. Vickey Boeriu, G. H. A. Viehhauser, S. Viel, R. Vigne, M. Villa, M. Villaplana Perez, E. Vilucchi, M. G. Vincter, V. B. Vinogradov, C. Vittori, I. Vivarelli, S. Vlachos, M. Vlasak, M. Vogel, P. Vokac, G. Volpi, M. Volpi, H. von der Schmitt, E. von Toerne, V. Vorobel, K. Vorobev, M. Vos, R. Voss, J. H. Vossebeld, N. Vranjes, M. Vranjes Milosavljevic, V. Vrba, M. Vreeswijk, R. Vuillermet, I. Vukotic, Z. Vykydal, P. Wagner, W. Wagner, H. Wahlberg, S. Wahrmund, J. Wakabayashi, J. Walder, R. Walker, W. Walkowiak, V. Wallangen, C. Wang, C. Wang, F. Wang, H. Wang, H. Wang, J. Wang, J. Wang, K. Wang, R. Wang, S. M. Wang, T. Wang, T. Wang, X. Wang, C. Wanotayaroj, A. Warburton, C. P. Ward, D. R. Wardrope, A. Washbrook, P. M. Watkins, A. T. Watson, I. J. Watson, M. F. Watson, G. Watts, S. Watts, B. M. Waugh, S. Webb, M. S. Weber, S. W. Weber, J. S. Webster, A. R. Weidberg, B. Weinert, J. Weingarten, C. Weiser, H. Weits, P. S. Wells, T. Wenaus, T. Wengler, S. Wenig, N. Wermes, M. Werner, P. Werner, M. Wessels, J. Wetter, K. Whalen, N. L. Whallon, A. M. Wharton, A. White, M. J. White, R. White, S. White, D. Whiteson, F. J. Wickens, W. Wiedenmann, M. Wielers, P. Wienemann, C. Wiglesworth, L. A. M. Wiik-Fuchs, A. Wildauer, H. G. Wilkens, H. H. Williams, S. Williams, C. Willis, S. Willocq, J. A. Wilson, I. Wingerter-Seez, F. Winklmeier, O. J. Winston, B. T. Winter, M. Wittgen, J. Wittkowski, S. J. Wollstadt, M. W. Wolter, H. Wolters, B. K. Wosiek, J. Wotschack, M. J. Woudstra, K. W. Wozniak, M. Wu, M. Wu, S. L. Wu, X. Wu, Y. Wu, T. R. Wyatt, B. M. Wynne, S. Xella, D. Xu, L. Xu, B. Yabsley, S. Yacoob, R. Yakabe, D. Yamaguchi, Y. Yamaguchi, A. Yamamoto, S. Yamamoto, T. Yamanaka, K. Yamauchi, Y. Yamazaki, Z. Yan, H. Yang, H. Yang, Y. Yang, Z. Yang, W-M. Yao, Y. C. Yap, Y. Yasu, E. Yatsenko, K. H. Yau Wong, J. Ye, S. Ye, I. Yeletskikh, A. L. Yen, E. Yildirim, K. Yorita, R. Yoshida, K. Yoshihara, C. Young, C. J. S. Young, S. Youssef, D. R. Yu, J. Yu, J. M. Yu, J. Yu, L. Yuan, S. P. Y. Yuen, I. Yusuff, B. Zabinski, R. Zaidan, A. M. Zaitsev, N. Zakharchuk, J. Zalieckas, A. Zaman, S. Zambito, L. Zanello, D. Zanzi, C. Zeitnitz, M. Zeman, A. Zemla, J. C. Zeng, Q. Zeng, K. Zengel, O. Zenin, T. Ženiš, D. Zerwas, D. Zhang, F. Zhang, G. Zhang, H. Zhang, J. Zhang, L. Zhang, R. Zhang, R. Zhang, X. Zhang, Z. Zhang, X. Zhao, Y. Zhao, Z. Zhao, A. Zhemchugov, J. Zhong, B. Zhou, C. Zhou, L. Zhou, L. Zhou, M. Zhou, N. Zhou, C. G. Zhu, H. Zhu, J. Zhu, Y. Zhu, X. Zhuang, K. Zhukov, A. Zibell, D. Zieminska, N. I. Zimine, C. Zimmermann, S. Zimmermann, Z. Zinonos, M. Zinser, M. Ziolkowski, L. Živković, G. Zobernig, A. Zoccoli, M. zur Nedden, G. Zurzolo, L. Zwalinski, ATLAS Collaboration

**Affiliations:** 1 Department of Physics, University of Adelaide, Adelaide, Australia; 2 Physics Department, SUNY Albany, Albany, NY USA; 3 Department of Physics, University of Alberta, Edmonton, AB Canada; 4 Department of Physics, Ankara University, Ankara, Turkey; 5 Istanbul Aydin University, Istanbul, Turkey; 6 Division of Physics, TOBB University of Economics and Technology, Ankara, Turkey; 7 LAPP, CNRS/IN2P3 and Université Savoie Mont Blanc, Annecy-le-Vieux, France; 8 High Energy Physics Division, Argonne National Laboratory, Argonne, IL USA; 9 Department of Physics, University of Arizona, Tucson, AZ USA; 10 Department of Physics, The University of Texas at Arlington, Arlington, TX USA; 11 Physics Department, University of Athens, Athens, Greece; 12 Physics Department, National Technical University of Athens, Zografou, Greece; 13 Department of Physics, The University of Texas at Austin, Austin, TX USA; 14 Institute of Physics, Azerbaijan Academy of Sciences, Baku, Azerbaijan; 15 Institut de Física d’Altes Energies (IFAE), The Barcelona Institute of Science and Technology, Barcelona, Spain; 16 Institute of Physics, University of Belgrade, Belgrade, Serbia; 17 Department for Physics and Technology, University of Bergen, Bergen, Norway; 18 Physics Division, Lawrence Berkeley National Laboratory and University of California, Berkeley, CA USA; 19 Department of Physics, Humboldt University, Berlin, Germany; 20 Albert Einstein Center for Fundamental Physics and Laboratory for High Energy Physics, University of Bern, Bern, Switzerland; 21 School of Physics and Astronomy, University of Birmingham, Birmingham, UK; 22 Department of Physics, Bogazici University, Istanbul, Turkey; 23 Department of Physics Engineering, Gaziantep University, Gaziantep, Turkey; 24 Faculty of Engineering and Natural Sciences, Istanbul Bilgi University, Istanbul, Turkey; 25 Faculty of Engineering and Natural Sciences, Bahcesehir University, Istanbul, Turkey; 26 Centro de Investigaciones, Universidad Antonio Narino, Bogotá, Colombia; 27 INFN Sezione di Bologna, Bologna, Italy; 28 Dipartimento di Fisica e Astronomia, Università di Bologna, Bologna, Italy; 29 Physikalisches Institut, University of Bonn, Bonn, Germany; 30 Department of Physics, Boston University, Boston, MA USA; 31 Department of Physics, Brandeis University, Waltham, MA USA; 32 Universidade Federal do Rio De Janeiro COPPE/EE/IF, Rio de Janeiro, Brazil; 33 Electrical Circuits Department, Federal University of Juiz de Fora (UFJF), Juiz de Fora, Brazil; 34 Federal University of Sao Joao del Rei (UFSJ), Sao Joao del Rei, Brazil; 35 Instituto de Fisica, Universidade de Sao Paulo, Sao Paulo, Brazil; 36 Physics Department, Brookhaven National Laboratory, Upton, NY USA; 37 Transilvania University of Brasov, Brasov, Romania; 38 National Institute of Physics and Nuclear Engineering, Bucharest, Romania; 39 Physics Department, National Institute for Research and Development of Isotopic and Molecular Technologies, Cluj Napoca, Romania; 40 University Politehnica Bucharest, Bucharest, Romania; 41 West University in Timisoara, Timisoara, Romania; 42 Departamento de Física, Universidad de Buenos Aires, Buenos Aires, Argentina; 43 Cavendish Laboratory, University of Cambridge, Cambridge, UK; 44 Department of Physics, Carleton University, Ottawa, ON Canada; 45 CERN, Geneva, Switzerland; 46 Enrico Fermi Institute, University of Chicago, Chicago, IL USA; 47 Departamento de Física, Pontificia Universidad Católica de Chile, Santiago, Chile; 48 Departamento de Física, Universidad Técnica Federico Santa María, Valparaiso, Chile; 49 Institute of High Energy Physics, Chinese Academy of Sciences, Beijing, China; 50 Department of Modern Physics, University of Science and Technology of China, Hefei, Anhui China; 51 Department of Physics, Nanjing University, Nanjing, Jiangsu China; 52 School of Physics, Shandong University, Jinan, Shandong China; 53 Shanghai Key Laboratory for Particle Physics and Cosmology, Department of Physics and Astronomy, Shanghai Jiao Tong University (also affiliated with PKU-CHEP), Shanghai, China; 54 Physics Department, Tsinghua University, Beijing, 100084 China; 55 Laboratoire de Physique Corpusculaire, Clermont Université and Université Blaise Pascal and CNRS/IN2P3, Clermont-Ferrand, France; 56 Nevis Laboratory, Columbia University, Irvington, NY USA; 57 Niels Bohr Institute, University of Copenhagen, Kobenhavn, Denmark; 58 INFN Gruppo Collegato di Cosenza, Laboratori Nazionali di Frascati, Frascati, Italy; 59 Dipartimento di Fisica, Università della Calabria, Rende, Italy; 60 Faculty of Physics and Applied Computer Science, AGH University of Science and Technology, Krakow, Poland; 61 Marian Smoluchowski Institute of Physics, Jagiellonian University, Krakow, Poland; 62 Institute of Nuclear Physics, Polish Academy of Sciences, Krakow, Poland; 63 Physics Department, Southern Methodist University, Dallas, TX USA; 64 Physics Department, University of Texas at Dallas, Richardson, TX USA; 65 DESY, Hamburg and Zeuthen, Hamburg, Germany; 66 Institut für Experimentelle Physik IV, Technische Universität Dortmund, Dortmund, Germany; 67 Institut für Kern- und Teilchenphysik, Technische Universität Dresden, Dresden, Germany; 68 Department of Physics, Duke University, Durham, NC USA; 69 SUPA-School of Physics and Astronomy, University of Edinburgh, Edinburgh, UK; 70 INFN Laboratori Nazionali di Frascati, Frascati, Italy; 71 Fakultät für Mathematik und Physik, Albert-Ludwigs-Universität, Freiburg, Germany; 72 Section de Physique, Université de Genève, Geneva, Switzerland; 73 INFN Sezione di Genova, Genoa, Italy; 74 Dipartimento di Fisica, Università di Genova, Genoa, Italy; 75 E. Andronikashvili Institute of Physics, Iv. Javakhishvili Tbilisi State University, Tbilisi, Georgia; 76 High Energy Physics Institute, Tbilisi State University, Tbilisi, Georgia; 77 II Physikalisches Institut, Justus-Liebig-Universität Giessen, Giessen, Germany; 78 SUPA-School of Physics and Astronomy, University of Glasgow, Glasgow, UK; 79 II Physikalisches Institut, Georg-August-Universität, Göttingen, Germany; 80 Laboratoire de Physique Subatomique et de Cosmologie, Université Grenoble-Alpes, CNRS/IN2P3, Grenoble, France; 81 Department of Physics, Hampton University, Hampton, VA USA; 82 Laboratory for Particle Physics and Cosmology, Harvard University, Cambridge, MA USA; 83 Kirchhoff-Institut für Physik, Ruprecht-Karls-Universität Heidelberg, Heidelberg, Germany; 84 Physikalisches Institut, Ruprecht-Karls-Universität Heidelberg, Heidelberg, Germany; 85 ZITI Institut für technische Informatik, Ruprecht-Karls-Universität Heidelberg, Mannheim, Germany; 86 Faculty of Applied Information Science, Hiroshima Institute of Technology, Hiroshima, Japan; 87 Department of Physics, The Chinese University of Hong Kong, Shatin, NT Hong Kong; 88 Department of Physics, The University of Hong Kong, Hong Kong, Hong Kong; 89 Department of Physics, The Hong Kong University of Science and Technology, Clear Water Bay, Kowloon, Hong Kong, China; 90 Department of Physics, Indiana University, Bloomington, IL USA; 91 Institut für Astro- und Teilchenphysik, Leopold-Franzens-Universität, Innsbruck, Austria; 92 University of Iowa, Iowa City, IA USA; 93 Department of Physics and Astronomy, Iowa State University, Ames, IA USA; 94 Joint Institute for Nuclear Research, JINR Dubna, Dubna, Russia; 95 KEK, High Energy Accelerator Research Organization, Tsukuba, Japan; 96 Graduate School of Science, Kobe University, Kobe, Japan; 97 Faculty of Science, Kyoto University, Kyoto, Japan; 98 Kyoto University of Education, Kyoto, Japan; 99 Department of Physics, Kyushu University, Fukuoka, Japan; 100 Instituto de Física La Plata, Universidad Nacional de La Plata and CONICET, La Plata, Argentina; 101 Physics Department, Lancaster University, Lancaster, UK; 102 INFN Sezione di Lecce, Lecce, Italy; 103 Dipartimento di Matematica e Fisica, Università del Salento, Lecce, Italy; 104 Oliver Lodge Laboratory, University of Liverpool, Liverpool, UK; 105 Department of Physics, Jožef Stefan Institute and University of Ljubljana, Ljubljana, Slovenia; 106 School of Physics and Astronomy, Queen Mary University of London, London, UK; 107 Department of Physics, Royal Holloway University of London, Surrey, UK; 108 Department of Physics and Astronomy, University College London, London, UK; 109 Louisiana Tech University, Ruston, LA USA; 110 Laboratoire de Physique Nucléaire et de Hautes Energies, UPMC and Université Paris-Diderot and CNRS/IN2P3, Paris, France; 111 Fysiska institutionen, Lunds universitet, Lund, Sweden; 112 Departamento de Fisica Teorica C-15, Universidad Autonoma de Madrid, Madrid, Spain; 113 Institut für Physik, Universität Mainz, Mainz, Germany; 114 School of Physics and Astronomy, University of Manchester, Manchester, UK; 115 CPPM, Aix-Marseille Université and CNRS/IN2P3, Marseille, France; 116 Department of Physics, University of Massachusetts, Amherst, MA USA; 117 Department of Physics, McGill University, Montreal, QC Canada; 118 School of Physics, University of Melbourne, Melbourne, VIC Australia; 119 Department of Physics, The University of Michigan, Ann Arbor, MI USA; 120 Department of Physics and Astronomy, Michigan State University, East Lansing, MI USA; 121 INFN Sezione di Milano, Milan, Italy; 122 Dipartimento di Fisica, Università di Milano, Milan, Italy; 123 B.I. Stepanov Institute of Physics, National Academy of Sciences of Belarus, Minsk, Republic of Belarus; 124 National Scientific and Educational Centre for Particle and High Energy Physics, Minsk, Republic of Belarus; 125 Group of Particle Physics, University of Montreal, Montreal, QC Canada; 126 P.N. Lebedev Physical Institute of the Russian, Academy of Sciences, Moscow, Russia; 127 Institute for Theoretical and Experimental Physics (ITEP), Moscow, Russia; 128 National Research Nuclear University MEPhI, Moscow, Russia; 129 D.V. Skobeltsyn Institute of Nuclear Physics, M.V. Lomonosov Moscow State University, Moscow, Russia; 130 Fakultät für Physik, Ludwig-Maximilians-Universität München, Munich, Germany; 131 Max-Planck-Institut für Physik (Werner-Heisenberg-Institut), Munich, Germany; 132 Nagasaki Institute of Applied Science, Nagasaki, Japan; 133 Graduate School of Science and Kobayashi-Maskawa Institute, Nagoya University, Nagoya, Japan; 134 INFN Sezione di Napoli, Naples, Italy; 135 Dipartimento di Fisica, Università di Napoli, Naples, Italy; 136 Department of Physics and Astronomy, University of New Mexico, Albuquerque, NM USA; 137 Institute for Mathematics, Astrophysics and Particle Physics, Radboud University Nijmegen/Nikhef, Nijmegen, The Netherlands; 138 Nikhef National Institute for Subatomic Physics and University of Amsterdam, Amsterdam, The Netherlands; 139 Department of Physics, Northern Illinois University, DeKalb, IL USA; 140 Budker Institute of Nuclear Physics, SB RAS, Novosibirsk, Russia; 141 Department of Physics, New York University, New York, NY USA; 142 Ohio State University, Columbus, OH USA; 143 Faculty of Science, Okayama University, Okayama, Japan; 144 Homer L. Dodge Department of Physics and Astronomy, University of Oklahoma, Norman, OK USA; 145 Department of Physics, Oklahoma State University, Stillwater, OK USA; 146 Palacký University, RCPTM, Olomouc, Czech Republic; 147 Center for High Energy Physics, University of Oregon, Eugene, OR USA; 148 LAL, Univ. Paris-Sud, CNRS/IN2P3, Université Paris-Saclay, Orsay, France; 149 Graduate School of Science, Osaka University, Osaka, Japan; 150 Department of Physics, University of Oslo, Oslo, Norway; 151 Department of Physics, Oxford University, Oxford, UK; 152 INFN Sezione di Pavia, Pavia, Italy; 153 Dipartimento di Fisica, Università di Pavia, Pavia, Italy; 154 Department of Physics, University of Pennsylvania, Philadelphia, PA USA; 155 National Research Centre “Kurchatov Institute” B.P. Konstantinov Petersburg Nuclear Physics Institute, St. Petersburg, Russia; 156 INFN Sezione di Pisa, Pisa, Italy; 157 Dipartimento di Fisica E. Fermi, Università di Pisa, Pisa, Italy; 158 Department of Physics and Astronomy, University of Pittsburgh, Pittsburgh, PA USA; 159 Laboratório de Instrumentação e Física Experimental de Partículas-LIP, Lisbon, Portugal; 160 Faculdade de Ciências, Universidade de Lisboa, Lisbon, Portugal; 161 Department of Physics, University of Coimbra, Coimbra, Portugal; 162 Centro de Física Nuclear da Universidade de Lisboa, Lisbon, Portugal; 163 Departamento de Fisica, Universidade do Minho, Braga, Portugal; 164 Departamento de Fisica Teorica y del Cosmos and CAFPE, Universidad de Granada, Granada, Spain; 165 Dep Fisica and CEFITEC of Faculdade de Ciencias e Tecnologia, Universidade Nova de Lisboa, Caparica, Portugal; 166 Institute of Physics, Academy of Sciences of the Czech Republic, Prague, Czech Republic; 167 Czech Technical University in Prague, Prague, Czech Republic; 168 Faculty of Mathematics and Physics, Charles University in Prague, Prague, Czech Republic; 169 State Research Center Institute for High Energy Physics (Protvino), NRC KI, Moscow, Russia; 170 Particle Physics Department, Rutherford Appleton Laboratory, Didcot, UK; 171 INFN Sezione di Roma, Rome, Italy; 172 Dipartimento di Fisica, Sapienza Università di Roma, Rome, Italy; 173 INFN Sezione di Roma Tor Vergata, Rome, Italy; 174 Dipartimento di Fisica, Università di Roma Tor Vergata, Rome, Italy; 175 INFN Sezione di Roma Tre, Rome, Italy; 176 Dipartimento di Matematica e Fisica, Università Roma Tre, Rome, Italy; 177 Faculté des Sciences Ain Chock, Réseau Universitaire de Physique des Hautes Energies-Université Hassan II, Casablanca, Morocco; 178 Centre National de l’Energie des Sciences Techniques Nucleaires, Rabat, Morocco; 179 Faculté des Sciences Semlalia, Université Cadi Ayyad, LPHEA-Marrakech, Marrakech, Morocco; 180 Faculté des Sciences, Université Mohamed Premier and LPTPM, Oujda, Morocco; 181 Faculté des Sciences, Université Mohammed V, Rabat, Morocco; 182 DSM/IRFU (Institut de Recherches sur les Lois Fondamentales de l’Univers), CEA Saclay (Commissariat à l’Energie Atomique et aux Energies Alternatives), Gif-sur-Yvette, France; 183 Santa Cruz Institute for Particle Physics, University of California Santa Cruz, Santa Cruz, CA USA; 184 Department of Physics, University of Washington, Seattle, WA USA; 185 Department of Physics and Astronomy, University of Sheffield, Sheffield, UK; 186 Department of Physics, Shinshu University, Nagano, Japan; 187 Fachbereich Physik, Universität Siegen, Siegen, Germany; 188 Department of Physics, Simon Fraser University, Burnaby, BC Canada; 189 SLAC National Accelerator Laboratory, Stanford, CA USA; 190 Faculty of Mathematics, Physics and Informatics, Comenius University, Bratislava, Slovakia; 191 Department of Subnuclear Physics, Institute of Experimental Physics of the Slovak Academy of Sciences, Kosice, Slovak Republic; 192 Department of Physics, University of Cape Town, Cape Town, South Africa; 193 Department of Physics, University of Johannesburg, Johannesburg, South Africa; 194 School of Physics, University of the Witwatersrand, Johannesburg, South Africa; 195 Department of Physics, Stockholm University, Stockholm, Sweden; 196 The Oskar Klein Centre, Stockholm, Sweden; 197 Physics Department, Royal Institute of Technology, Stockholm, Sweden; 198 Departments of Physics and Astronomy and Chemistry, Stony Brook University, Stony Brook, NY USA; 199 Department of Physics and Astronomy, University of Sussex, Brighton, UK; 200 School of Physics, University of Sydney, Sydney, Australia; 201 Institute of Physics, Academia Sinica, Taipei, Taiwan; 202 Department of Physics, Technion: Israel Institute of Technology, Haifa, Israel; 203 Raymond and Beverly Sackler School of Physics and Astronomy, Tel Aviv University, Tel Aviv, Israel; 204 Department of Physics, Aristotle University of Thessaloniki, Thessaloniki, Greece; 205 International Center for Elementary Particle Physics and Department of Physics, The University of Tokyo, Tokyo, Japan; 206 Graduate School of Science and Technology, Tokyo Metropolitan University, Tokyo, Japan; 207 Department of Physics, Tokyo Institute of Technology, Tokyo, Japan; 208 Department of Physics, University of Toronto, Toronto, ON Canada; 209 TRIUMF, Vancouver, BC Canada; 210 Department of Physics and Astronomy, York University, Toronto, ON Canada; 211 Faculty of Pure and Applied Sciences, and Center for Integrated Research in Fundamental Science and Engineering, University of Tsukuba, Tsukuba, Japan; 212 Department of Physics and Astronomy, Tufts University, Medford, MA USA; 213 Department of Physics and Astronomy, University of California Irvine, Irvine, CA USA; 214 INFN Gruppo Collegato di Udine, Sezione di Trieste, Udine, Italy; 215 ICTP, Trieste, Italy; 216 Dipartimento di Chimica Fisica e Ambiente, Università di Udine, Udine, Italy; 217 Department of Physics and Astronomy, University of Uppsala, Uppsala, Sweden; 218 Department of Physics, University of Illinois, Urbana, IL USA; 219 Instituto de Fisica Corpuscular (IFIC) and Departamento de Fisica Atomica, Molecular y Nuclear and Departamento de Ingeniería Electrónica and Instituto de Microelectrónica de Barcelona (IMB-CNM), University of Valencia and CSIC, Valencia, Spain; 220 Department of Physics, University of British Columbia, Vancouver, BC Canada; 221 Department of Physics and Astronomy, University of Victoria, Victoria, BC Canada; 222 Department of Physics, University of Warwick, Coventry, UK; 223 Waseda University, Tokyo, Japan; 224 Department of Particle Physics, The Weizmann Institute of Science, Rehovot, Israel; 225 Department of Physics, University of Wisconsin, Madison, WI USA; 226 Fakultät für Physik und Astronomie, Julius-Maximilians-Universität, Würzburg, Germany; 227 Fakultät für Mathematik und Naturwissenschaften, Fachgruppe Physik, Bergische Universität Wuppertal, Wuppertal, Germany; 228 Department of Physics, Yale University, New Haven, CT USA; 229 Yerevan Physics Institute, Yerevan, Armenia; 230 Centre de Calcul de l’Institut National de Physique Nucléaire et de Physique des Particules (IN2P3), Villeurbanne, France; 231 CERN, 1211 Geneva 23, Switzerland

## Abstract

The results of a search for gluinos in final states with an isolated electron or muon, multiple jets and large missing transverse momentum using proton–proton collision data at a centre-of-mass energy of $\sqrt{s} = 13 \mathrm{{\ Te V}}$ are presented. The dataset used was recorded in 2015 by the ATLAS experiment at the Large Hadron Collider and corresponds to an integrated luminosity of 3.2 fb$^{-1}$. Six signal selections are defined that best exploit the signal characteristics. The data agree with the Standard Model background expectation in all six signal selections, and the largest deviation is a 2.1 standard deviation excess. The results are interpreted in a simplified model where pair-produced gluinos decay via the lightest chargino to the lightest neutralino. In this model, gluinos are excluded up to masses of approximately 1.6 Te V depending on the mass spectrum of the simplified model, thus surpassing the limits of previous searches.

## Introduction

Supersymmetry (SUSY) [1–6] is a theoretical framework of physics beyond the Standard Model (SM) that predicts for each SM particle the existence of a supersymmetric partner differing by half a unit of spin. The partner particles of the SM fermions (quarks and leptons) are the scalar squarks ($\tilde{q}$) and sleptons ($\tilde{\ell }$). In the boson sector, the supersymmetric partner of the gluon is the fermionic gluino ($\tilde{g}$), whereas the supersymmetric partners of the Higgs (higgsinos) and the electroweak gauge bosons (winos and bino) mix to form charged and neutral mass eigenstates called charginos ($\tilde{\chi }^{\pm }_{1,2}$) and neutralinos ($\tilde{\chi }^{0}_{1,2,3,4}$). In the Minimal Supersymmetric extension of the Standard Model (MSSM) [7, 8] two scalar Higgs doublets along with their Higgsino partners are predicted. SUSY addresses the SM hierarchy problem [9–12] provided that the masses of at least some of the supersymmetric particles (most notably the higgsinos, the top squarks and the gluinos) are near the Te V scale.

In R-parity-conserving SUSY [13], gluinos might be pair-produced at the Large Hadron Collider (LHC) via the strong interaction and decay either directly or via intermediate states to the lightest supersymmetric particle (LSP). The LSP is stable and is assumed to be only weakly interacting, making it a candidate for dark matter [14, 15].

This paper considers a SUSY-inspired model where pair-produced gluinos decay via the lightest chargino ($\tilde{\chi }^{\pm }_{1}$) to the LSP, which is assumed to be the lightest neutralino (${\tilde{\chi }^0_1}$). The three-body decay of the gluino to the chargino proceeds via $\tilde{g} \rightarrow q\bar{q'} \tilde{\chi }^\pm _1$. The chargino decays to the LSP by emitting an on- or off-shell *W* boson, depending on the available phase space. In the MSSM this decay chain is realised when the gluino decays via a virtual squark that is the partner of the left-handed SM quark, to the chargino with a dominant wino component. In the MSSM the mass of the chargino is independent of the mass of the gluino.

The experimental signature characterising this search consists of a lepton (electron or muon), several jets, and missing transverse momentum (${\varvec{p}}_{\mathrm {T}}^\mathrm {miss}$ with magnitude $E_\mathrm{{T}}^\mathrm{{miss}}$) from the undetectable neutralinos and neutrino(s). The analysis is based on two complementary sets of search channels. The first set requires a low transverse momentum ($p_{\mathrm {T}}$) lepton ($7/6< p_{\mathrm {T}}(e/\mu ) < 35 \mathrm {\ Ge V}$), and is referred to as the soft-lepton channel, while the second set requires a high-$p_{\mathrm {T}}$ lepton ($p_{\mathrm {T}}(e/\mu ) > 35 \mathrm {\ Ge V}$) and is referred to as the hard-lepton channel. The two sets target SUSY models with small and large mass differences between the predicted supersymmetric particles, respectively. The search uses the ATLAS data collected in proton–proton LHC collisions in 2015 corresponding to an integrated luminosity of 3.2 fb$^{-1}$at a centre-of-mass energy of 13 Te V.

The analysis extends previous ATLAS searches with similar event selections [16] which were performed with data collected during the first data-taking campaign between 2010 and 2012 (LHC Run 1) at a centre-of-mass energy of up to 8 Te V. The results of all Run 1 ATLAS searches targeting squark and gluino pair production are summarised in Ref. [17]. The CMS Collaboration has performed similar searches for gluinos with decays via intermediate supersymmetric particles in Run 1 [18, 19] and Run 2 [20].

This paper is structured as follows. After a brief description of the ATLAS detector in Sect. 2, the simulated data samples for the background and signal processes used in the analysis as well as the dataset and the trigger strategy are detailed in Sect. 3. The reconstructed objects and quantities used in the analysis are described in Sect. 4 and the event selection is presented in Sect. 5. The background estimation and the systematic uncertainties associated with the expected event yields are discussed in Sects. 6 and 7, respectively, while details of the statistical interpretation are given in Sect. 8. Finally, the results of the analysis are presented in Sect. 9 and are followed by a conclusion.

## ATLAS detector

ATLAS [21] is a general-purpose detector with a forward-backward symmetric design that provides almost full solid angle coverage around the interaction point.^1^ The main components of ATLAS are the inner detector (ID), which is surrounded by a superconducting solenoid providing a 2 T axial magnetic field, the calorimeter system, and the muon spectrometer (MS), which is immersed in a magnetic field generated by three large superconducting toroidal magnets. The ID provides track reconstruction within $|\eta | < 2.5$, employing pixel detectors close to the beam pipe, silicon microstrip detectors at intermediate radii, and a straw-tube tracker with particle identification capabilities based on transition radiation at radii up to 1080 mm. The innermost pixel detector layer, the insertable B-layer [22], was added during the shutdown between LHC Run 1 and Run 2, at a radius of 33 mm around a new, narrower, beam pipe. The calorimeters cover $|\eta | < 4.9$, the forward region ($3.2< |\eta | < 4.9$) being instrumented with a liquid-argon (LAr) calorimeter for both the electromagnetic and the hadronic measurements. In the central region, a lead/LAr electromagnetic calorimeter covers $|\eta | < 3.2$, while the hadronic calorimeter uses two different detector technologies, with scintillator tiles ($|\eta | < 1.7$) or LAr ($1.5< |\eta | < 3.2$) as the active medium. The MS consists of three layers of precision tracking chambers providing coverage over $|\eta | < 2.7$, while dedicated fast chambers allow triggering over $|\eta | < 2.4$. The ATLAS trigger system (developed from Ref. [23]) consists of a hardware-based first-level trigger and a software-based high-level trigger.

## Simulated event samples and data samples

**Fig. 1:**
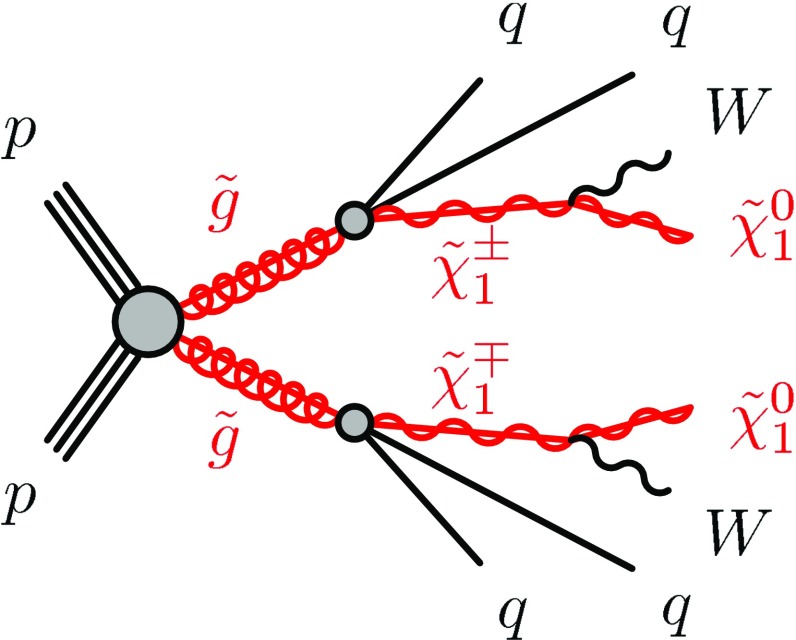
The decay topology of the signal model considered in this search

The signal model considered in this search is a simplified model [24–26] that has been used in previous similar ATLAS searches [16]. In this model, exclusive pair-production of gluinos is assumed. The gluinos decay via an intermediate chargino, here the lightest chargino $\tilde{\chi }^\pm _1$, into the lightest supersymmetric particle, the lightest neutralino ${\tilde{\chi }^0_1}$. The branching ratio of each supersymmetric particle decay considered is assumed to be 100 %. Other supersymmetric particles not entering the decay chain described are not considered in this simplified model and their masses are set to high values. The gluino decay is assumed to proceed only via virtual first- and second- generation quarks, hence no bottom or top quarks are produced in the simplified model. The free parameters of the model are the masses of the gluino ($m_{\tilde{g}}$), the chargino ($m_{\tilde{\chi }^\pm _1}$), and the neutralino ($m_{\tilde{\chi }^{0}_{1}}$). Two types of scenarios are considered: in the first type, the mass of the neutralino is fixed to $60 \mathrm {\ Ge V}$, and the sensitivity is assessed as a function of the gluino mass and a mass-ratio parameter defined as $x = (m_{\tilde{\chi }_{1}^{\pm }} - m_{\tilde{\chi }^{0}_{1}})/(m_{\tilde{g}} - m_{\tilde{\chi }^{0}_{1}})$. In the second type, $m_{\tilde{g}}$ and $m_{{\tilde{\chi }^0_1}}$ are free parameters, while $m_{\tilde{\chi }^\pm _1}$ is set to $m_{\tilde{\chi }^\pm _1} = (m_{\tilde{g}} + m_{{\tilde{\chi }^0_1}})/2$. The decay topology of the simplified model is illustrated in Fig. 1.

The signal samples are generated using MG5_aMC@NLO 2.2.2 [27] with up to two extra partons in the matrix element, interfaced to Pythia 8.186 [28] for parton showers and hadronisation. For the combination of the matrix element and the parton shower the CKKW-L matching scheme [29] is applied with a scale parameter that is set to a quarter of the mass of the gluino. The ATLAS A14 [30] set of tuned parameters (tune) for the underlying event is used together with the NNPDF2.3 LO [31] parton distribution function (PDF) set. The EvtGen v1.2.0 program [32] is used to describe the properties of the bottom and charm hadron decays in the signal samples.

The signal cross-sections are calculated at next-to-leading order (NLO) in the strong coupling constant, adding the resummation of soft gluon emission at next-to-leading-logarithmic accuracy (NLL) [33–37]. The nominal cross-section and its uncertainty are taken from an envelope of cross-section predictions using different PDF sets and factorisation and renormalisation scales [38, 39].

**Table 1:** Simulated background event samples: the corresponding generator, parton shower, cross-section normalisation, PDF set and underlying-event tune are shown

Physics process	Generator	Parton shower	Cross-section normalisation	PDF set	Tune
$W(\rightarrow \ell \nu )$ + jets	Sherpa 2.1.1 [40]	Sherpa 2.1.1	NNLO	NLO CT10	Sherpa default
$Z/\gamma ^{*}(\rightarrow \ell \ell )$ + jets	Sherpa 2.1.1	Sherpa 2.1.1	NNLO	NLO CT10	Sherpa default
$t\bar{t}$	powheg-box v2	Pythia 6.428 [41]	NNLO+NNLL	NLO CT10	Perugia2012 [42]
Single-top					
(*t*-channel)	powheg-box v1	Pythia 6.428	NLO	NLO CT10f4	Perugia2012
Single-top					
(*s*- and *Wt*-channel)	powheg-box v2	Pythia 6.428	NLO	NLO CT10	Perugia2012
$t\bar{t}+W/Z/WW$	MG5_aMC@NLO 2.2.2	Pythia 8.186	NLO	NNPDF2.3LO	A14
*WW*, *WZ* and *ZZ*	Sherpa 2.1.1	Sherpa 2.1.1	NLO	NLO CT10	Sherpa default

The simulated event samples for the SM backgrounds are summarised in Table 1, along with the PDFs and tunes used. Further samples are also used to assess systematic uncertainties, as explained in Sect. 7.

For the production of $t\bar{t}$ and single top quarks in the *Wt* and *s*-channel [43] the powheg-box v2 [44] generator with the CT10 [45] PDF sets in the matrix-element calculations is used. Electroweak *t*-channel single-top-quark events are generated using the powheg-box v1 generator. This generator uses the four-flavour scheme for the NLO matrix-element calculations together with the fixed four-flavour PDF set CT10f4. For all top-quark processes, top-quark spin correlations are preserved (for the single-top *t*-channel, top quarks are decayed using MadSpin [46]). The parton shower, fragmentation and the underlying event are simulated using Pythia 6.428 with the CTEQ6L1 [47] PDF set and the corresponding Perugia2012 tune (P2012) [42]. The top-quark mass is assumed to be $172.5 \mathrm {\ Ge V}$. The $t\bar{t}$ events are normalised to the NNLO+NNLL cross-sections. The single-top-quark events are normalised to the NLO cross-sections.

Events containing *W* or *Z* bosons with associated jets (*W*/*Z*+jets) [48] are simulated using the Sherpa 2.1.1 generator with massive *b* / *c*-quarks. Matrix elements are calculated for up to two partons at NLO and four partons at leading order (LO). The matrix elements are calculated using the Comix [49] and OpenLoops [50] generators and merged with the Sherpa 2.1.1 parton shower [51] using the ME+PS@NLO prescription [52]. The CT10 PDF set is used in conjunction with a dedicated parton-shower tuning developed by the Sherpa authors. The *W* / *Z*+jets events are normalised to their NNLO cross-sections [53].

The diboson samples [54] are generated with the same generator and PDF setup as the *W* / *Z*+jets samples described above. The diboson processes are simulated including final states with four charged leptons, three charged leptons and one neutrino, two charged leptons and two neutrinos, and one charged lepton and three neutrinos. The matrix elements contain all diagrams with four electroweak vertices. They are calculated for up to one parton ($4 \ell$, $2 \ell$+$2\nu$) or no additional partons ($3 \ell$+$1 \nu$, $1 \ell$+$3 \nu$) at NLO and up to three partons at LO. The diboson cross-sections are taken from the NLO generator used.

For the $t\bar{t}+W/Z/WW$ processes [55], all events are simulated using MG5_aMC@NLO 2.2.2 at LO interfaced to the Pythia 8.186 parton-shower model, with up to two ($t\bar{t}+W$), one ($t\bar{t}+Z$) or no ($t\bar{t}+WW$) extra partons included in the matrix element. The ATLAS underlying-event tune A14 is used together with the NNPDF2.3 LO PDF set. The events are normalised to their respective NLO cross-sections [56, 57].

The response of the detector to particles is modelled with a full ATLAS detector simulation [58] using Geant4 [59], or using a fast simulation [60] based on a parameterisation of the performance of the electromagnetic and hadronic calorimeters and on Geant4 elsewhere. All background (signal) samples are prepared using the full (fast) detector simulation.

All simulated samples are generated with a varying number of minimum-bias interactions (simulated using Pythia 8 with the MSTW2008LO PDF set [61] and the A2 tune [62]) overlaid on the hard-scattering event to model the multiple proton–proton interactions in the same and the nearby bunch crossings. Corrections are applied to the simulated samples to account for differences between data and simulation for trigger, identification and reconstruction efficiencies.

The proton–proton data analysed in this paper were collected by ATLAS in 2015 at a centre-of-mass energy of 13  Te V. During this period the instantaneous luminosity of the LHC reached $5.0 \times 10^{33}$ cm$^{-2}\mathrm {s}^{-1}$ with a mean number of additional *pp* interactions per bunch crossing of approximately 14. After application of data-quality requirements related to the beam and detector conditions, the total integrated luminosity amounts to 3.2 fb$^{-1}$, with an associated uncertainty of $\pm 5$ %. These values are derived following the same methodology as the one detailed in Ref. [63].

The data are collected using an $E_\mathrm{{T}}^\mathrm{{miss}}$ trigger with a threshold of $70 \mathrm {\ Ge V}$. This trigger is close to fully efficient after applying the requirement on the offline $E_\mathrm{{T}}^\mathrm{{miss}}$ to be larger than $200~\mathrm {\ Ge V}$.

## Object reconstruction and identification

The reconstructed primary vertex of an event is required to be consistent with the interaction region and to have at least two associated tracks with $p_{\mathrm {T}}> 400 \mathrm {\ Me V}$. When more than one such vertex is found, the vertex with the largest $\sum p_\mathrm {T}^2$ of the associated tracks is chosen.

In the analysis, a distinction is made between preselected reconstructed objects, which fulfil a set of basic criteria and are used in the $E_\mathrm{{T}}^\mathrm{{miss}}$ computation, and signal objects that enter the various control, validation and signal regions and are subject to more stringent requirements.

Jets are reconstructed from topological clusters in the calorimeters using the anti-$k_t$ algorithm with a radius parameter $R=0.4$ [64, 65]. Prior to jet reconstruction, clusters are calibrated to the electromagnetic scale response. Additional correction factors derived from simulation and data are applied to the measured jet energy to calibrate it to the particle level [65]. To mitigate the contributions from pile-up, the median energy density of all the jets in the event, multiplied by the jet area, is subtracted from the reconstructed jet energy [66, 67]. Preselected jets are required to have $p_{\mathrm {T}}> 20 \mathrm {\ Ge V}$ and $|\eta | < 4.5$. The contamination from cosmic rays, other sources of non-collision background and detector noise is suppressed using dedicated jet-quality criteria [68]: when such criteria are not fulfilled, the event is rejected.

Electron candidates are reconstructed using ID tracks matched to energy clusters in the electromagnetic calorimeter. They are identified according to the likelihood-based loose criteria [69]. Preselected electrons in the soft-lepton (hard-lepton) channel must satisfy $p_\text {T}>7 (10) \mathrm {\ Ge V}$ and $|\eta |<2.47$. When the angular separation $\Delta R=\sqrt{(\Delta y)^2+(\Delta \phi )^2}$ between an electron candidate and a preselected jet amounts to $0.2<\Delta R(e,\mathrm {jet})<0.4$, the jet is retained and the electron is rejected to remove electrons originating from *b*-hadron decays. Since all electrons are also reconstructed as jets, if $\Delta R(e,\mathrm {jet})<0.2$ the electron is kept and the jet is discarded. Finally, electron candidates with a $\Delta R(e,\mu )<0.01$ with respect to a preselected muon (defined below) are rejected and the muon is kept to suppress the contribution of electron candidates from muon bremsstrahlung and subsequent photon conversion.

Muon candidates are reconstructed by combining tracks formed in the ID and the MS sub-systems. The Medium identification criteria are applied, which offer good efficiency and purity for the selected muons [70]. Preselected muons in the soft-lepton (hard-lepton) channel are required to have $p_{\mathrm {T}}>6 (10) \mathrm {\ Ge V}$ and $|\eta |<2.4$. Muons with an angular separation of $\Delta R(\mu ,\mathrm {jet})<0.4$ with respect to the closest preselected jet are rejected, after the electron–jet overlap ambiguities are resolved. However, if the number of tracks with $p_{\mathrm {T}}> 500 \mathrm {\ Me V}$ associated with the jet is less than three the jet is discarded and the muon kept.

The $E_\mathrm{{T}}^\mathrm{{miss}}$ is calculated as the magnitude of the negative vector sum of the transverse momenta of identified and calibrated muons, electrons, jets and photons, in addition to the soft-track term. The soft-track term is defined as the vectorial sum of the ${\varvec{p}}_{\mathrm {T}}$ of all reconstructed tracks associated with the primary vertex that are not associated with the identified objects entering explicitly the $E_\mathrm{{T}}^\mathrm{{miss}}$ computation [71, 72].

Signal jets are required to have $p_{\mathrm {T}}>25 \mathrm {\ Ge V}$ and $|\eta |<2.8$. A likelihood discriminant, the jet-vertex tagger (JVT), is used to remove the residual contamination of pile-up jets. The JVT is constructed from track-based variables that are sensitive to the vertex of origin of the jet [73]. Jets with $p_{\mathrm {T}}<50 \mathrm {\ Ge V}$, $|\eta |<2.4$ and JVT score less than 0.64 are rejected.

Signal jets containing *b*-hadrons are identified using the MV2c20 algorithm [74] and are hereafter referred to as *b*-tagged jets. The MV2c20 algorithm uses as input the impact parameters of all associated tracks and any reconstructed secondary vertex. The requirement chosen in the analysis provides an inclusive *b*-tagging efficiency of 77 % in simulated $t\bar{t}$ events, along with a rejection factor of 140 for gluon and light-quark jets and of 4.5 for charm jets [74, 75].

Signal muons and electrons in the soft-lepton and hard-lepton channels are subject to an additional $p_{\mathrm {T}}<35 \mathrm {\ Ge V}$ or $p_{\mathrm {T}}\ge 35 \mathrm {\ Ge V}$ requirement, respectively. Electrons must satisfy likelihood-based tight criteria which are defined in Ref. [69]. In both channels, signal leptons must satisfy the GradientLoose [70] isolation requirements, which rely on the use of tracking-based and calorimeter-based variables and implement a set of $\eta$- and $p_{\mathrm {T}}$-dependent criteria. The efficiency for prompt leptons with transverse momentum $< 40$ Ge V to satisfy the GradientLoose requirements is measured to be about 95 % in *Z* $\rightarrow$ $\ell \ell$ events, progressively rising up to 99 % at 100 Ge V [70].

To enforce compatibility with the primary vertex, the distance $|z_0\cdot \mathrm {sin}(\theta )|$ is required to be less than $0.5~\mathrm {mm}$ for signal lepton tracks, where $z_0$ is the longitudinal impact parameter with respect to the primary-vertex position. Moreover, in the transverse plane the distance of closest approach of the lepton track to the proton beam line, divided by the corresponding uncertainty, must be less than three for muons and less than five for electrons.

Reconstruction, identification and isolation efficiencies in simulation, when applicable, are calibrated to data for all reconstructed objects.

## Event selection

Events selected by the trigger are further required to have a reconstructed primary vertex. An event is rejected if it contains a preselected jet which fails to satisfy the quality criteria designed to suppress non-collision backgrounds and detector noise [68]. Exactly one signal lepton is required in both the soft- and the hard-lepton channels. Any event with additional preselected leptons is vetoed to suppress the dilepton $t\bar{t}$, single-top (*Wt*-channel) and diboson backgrounds.

A dedicated optimisation study was performed to design signal region (SR) selection criteria and to maximise the signal sensitivity. Four hard-lepton signal regions and two soft-lepton signal regions are defined, targeting different mass hierarchy scenarios in the simplified model. The selection criteria used to define the signal regions are summarised in Table 2 for the soft-lepton channel and in Table 3 for the hard-lepton channel.

**Table 2:** Overview of the selection criteria for the soft-lepton signal regions. The symbol $p_{\mathrm {T}}^\ell$ refers to signal leptons

	**2-jet soft-lepton SR**	**5-jet soft-lepton SR**
$N_{\mathrm {lep}}$($p_{\mathrm {T}}^\ell$ $^{=e(\mu )} > 7(6)$ Ge V)	$= 1$	$= 1$
$p_{\mathrm {T}}^\ell$ $^{=e(\mu )}$ ( Ge V)	7(6)–35	7(6)–35
$N_{\mathrm {jet}}$	$\ge 2$	$\ge 5$
$p_{\mathrm {T}}^{\mathrm {jet}}$ ( Ge V)	> 180, 30	> 200, 200, 200, 30, 30
$E_\mathrm{{T}}^\mathrm{{miss}}$ ( Ge V)	$> 530$	$> 375$
$m_{\mathrm {T}}$ ( Ge V)	$> 100$	–
$E_\mathrm{{T}}^\mathrm{{miss}}$/$m_{\mathrm {eff}}^{\mathrm {inc}}$	$> 0.38$	–
$H_{\mathrm {T}}$ ( Ge V)	–	$> 1100$
Jet aplanarity	–	$> 0.02$

**Table 3:** Overview of the selection criteria for the hard-lepton signal regions. The symbol $p_{\mathrm {T}}^\ell$ refers to signal leptons. The mass-ratio parameter *x* used in the signal region labels is defined in Sect. 3

	**4-jet high-** *x* ** SR**	**4-jet low-** *x* ** SR**	**5-jet SR**	**6-jet SR**
$N_{\mathrm {lep}}$($p_{\mathrm {T}}^\ell$ $^{=e(\mu )} {>}10$ Ge V)	$= 1$	$= 1$	$= 1$	$= 1$
$p_{\mathrm {T}}^\ell$ $^{=e(\mu )}$ ( Ge V)	${>}35$	${>}35$	${>}35$	${>}35$
$N_{\mathrm {jet}}$	${\ge } 4$	${\ge } 4$	${\ge }5$	${\ge }6$
$p_{\mathrm {T}}^{\mathrm {jet}}$ ( Ge V)	${>}325, 30,... , 30$	${>}325, 150,... , 150$	${>}225, 50,... , 50$	${>}125, 30,... , 30$
$E_\mathrm{{T}}^\mathrm{{miss}}$ ( Ge V)	${>}200$	${>}200$	${>}250$	${>}250$
$m_{\mathrm {T}}$ ( Ge V)	${>}425$	${>}125$	${>}275$	${>}225$
$E_\mathrm{{T}}^\mathrm{{miss}}$/$m_{\mathrm {eff}}^{\mathrm {inc}}$	${>}0.3$	–	${>}0.1$	${>}0.2$
$m_{\mathrm {eff}}^{\mathrm {inc}}$ ( Ge V)	${>}1800$	${>}2000$	${>}1800$	${>}1000$
Jet aplanarity	–	${>}0.04$	${>}0.04$	${>}0.04$

The observables defined below are used in the event selection.

The transverse mass ($m_\mathrm{{T}}$) of the lepton and the ${\varvec{p}}_{\mathrm {T}}^\mathrm {miss}$ is defined as

1 $$\begin{aligned} m_{\mathrm {T}} = \sqrt{2 p_{\mathrm {T}}^{\ell } E_\mathrm{{T}}^\mathrm{{miss}}(1-\cos [\Delta \phi ({\varvec{p}}_{\mathrm {T}}^{\ell },{\varvec{p}}_{\mathrm {T}}^\mathrm {miss})])}, \end{aligned}$$

where $\Delta \phi ({\varvec{p}}_{\mathrm {T}}^{\ell },{\varvec{p}}_{\mathrm {T}}^\mathrm {miss})$ is the azimuthal angle between the lepton and the missing transverse momentum. This is used in the soft-lepton 2-jet signal region and all hard-lepton signal regions to reject *W*+jets and semileptonic $t\bar{t}$ events.

The inclusive effective mass ($m_\mathrm{{eff}}^\mathrm{{inc}}$) is the scalar sum of the $p_{\mathrm {T}}$ of the signal lepton and jets and the $E_\mathrm{{T}}^\mathrm{{miss}}$:

2 $$\begin{aligned} m_{\mathrm {eff}}^{\mathrm {inc}} = p_{\mathrm {T}}^{\ell } + \sum _{j=1}^{{N}_\mathrm {jet}} p_{\mathrm {T},j} + E_\mathrm{{T}}^\mathrm{{miss}}, \end{aligned}$$

where the index *j* runs over all the signal jets in the event with $p_{\mathrm {T}}> 30 \mathrm {\ Ge V}$. The inclusive effective mass provides good discrimination against SM backgrounds, without being too sensitive to the details of the SUSY cascade decay chain.

The transverse momentum scalar sum ($H_{\mathrm {T}}$) is defined as

3 $$\begin{aligned} H_{\mathrm {T}}= p_{\mathrm {T}}^{\ell } + \sum _{j=1}^{{N}_\mathrm {jet}}p_{\mathrm {T},j}, \end{aligned}$$

where the index *j* runs over all the signal jets in the event. The $H_{\mathrm {T}}$ variable is used to define the soft-lepton 5-jet signal region, as the many energetic jets in the signal model render this variable useful to separate signal from background.

The ratio $E_\mathrm{{T}}^\mathrm{{miss}}$/$m_\mathrm{{eff}}^{\mathrm {inc}}$ is used in both the soft- and the hard-lepton channels; it provides good discrimination power between signal and background with fake $E_\mathrm{{T}}^\mathrm{{miss}}$ due to instrumental effects.

Additional suppression of background processes is based on the aplanarity variable, which is defined as $\mathcal A$ = $\frac{3}{2}$ $\lambda _{3}$, where $\lambda _{3}$ is the smallest eigenvalue of the normalised momentum tensor of the jets [76]. Typical measured values lie in the range $0 \leqslant \mathcal{A} < 0.3$, with values near zero indicating relatively planar background-like events.

The hard-lepton 5-jet region targets scenarios with high gluino masses and low $\tilde{\chi }^{0}_{1}$ masses in models with the chargino mass $m_{\tilde{\chi }_{1}^{\pm }}$ chosen such that the mass-ratio parameter $x = 1/2$. Tight requirements on $m_{\mathrm {T}}$ and $m_\mathrm{{eff}}^{\mathrm {inc}}$ are applied. For the same set of models, the hard-lepton 6-jet region is designed to provide sensitivity to scenarios where the mass separation between the gluino and the neutralino is smaller. For this reason, the requirements on $m_{\mathrm {T}}$ and $m_\mathrm{{eff}}^{\mathrm {inc}}$ are relaxed with respect to the hard-lepton 5-jet region. Two distinct hard-lepton 4-jet regions are used, both designed to target models where the neutralino mass is fixed to 60 Ge V, while the gluino mass and the mass-ratio *x* vary. The 4-jet high-*x* region is designed for regions of the parameter space where the *W* boson produced in the chargino decay is significantly boosted, leading to high-$p_{\mathrm {T}}$ leptons. The main characteristics of signal events in the phase-space of this model are large $m_{\mathrm {T}}$ values and relatively soft jets emitted from the gluino decays. In the 4-jet low-*x* region, the *W* boson tends to be virtual while the jets from the gluino decays tend to have high $p_{\mathrm {T}}$ due to the large gluino–chargino mass difference. Therefore, the $m_{\mathrm {T}}$ requirement is relaxed and more stringent jet $p_{\mathrm {T}}$ requirements are imposed.

The soft-lepton channels focus on models with compressed mass spectra. The soft-lepton 2-jet region provides sensitivity to scenarios characterised by a relatively heavy neutralino and a small mass separation between the gluino, the chargino and the neutralino. Due to the small mass separation, most of the decay products tend to be low $p_{\mathrm {T}}$, or soft. Thus, a high-$p_{\mathrm {T}}$ initial-state radiation (ISR) jet recoiling against the rest of the event is required, in order to enhance the kinematic properties of the signal and to provide separation with respect to the backgrounds. The soft-lepton 5-jet region is designed to be sensitive to the configurations in parameter space with a large mass gap between the gluino and chargino and a small separation between $m_{\tilde{\chi }_{1}^{\pm }}$ and $m_{\tilde{\chi }^{0}_{1}}$. As a consequence, several energetic jets from the decay of the two gluinos to the charginos are expected, while the virtual *W* bosons produced in the decay of the charginos result in low-$p_{\mathrm {T}}$ jets and leptons.

## Background estimation

The two dominant background processes in final states with one isolated lepton, multiple jets and large missing transverse momentum are $t\bar{t}$ and *W*+jets. The differential distributions arising from these two background processes as predicted from simulation are simultaneously normalised to the number of data events observed in dedicated control regions (CR), through the fitting procedure explained in Sect. 8. The simulation is then used to extrapolate the measured background rates to the corresponding signal region.

The control regions are designed to have high purity in the process of interest, a small contamination from the signal model and enough events to result in a small statistical uncertainty in the background prediction. Moreover, they are designed to have kinematic properties resembling as closely as possible those of the signal regions, in order to provide good estimates of the kinematics of background processes there. This procedure limits the impact of potentially large systematic uncertainties in the expected yields.

Additional sources of background events are single-top events (*s*-channel, *t*-channel and associated production with a *W* boson), *Z*+jets and diboson processes (*WW*, *WZ*, *ZZ*, $W\gamma$, $Z\gamma$), and $t\bar{t}$ production in association with a *W* or a *Z* boson. Their contributions are estimated entirely using simulated event samples normalised to the most accurate theoretical cross-sections available.

The contribution from multi-jet processes with a misidentified lepton is found to be negligible once lepton isolation criteria and a stringent $E_\mathrm{{T}}^\mathrm{{miss}}$ requirement are imposed. A data-driven matrix method, following the implementation described in Ref. [16], confirms this background is consistent with zero. This is mainly a result of the improved lepton reconstruction and identification and the higher threshold on $E_\mathrm{{T}}^\mathrm{{miss}}$ with respect to the previous searches performed in this final state [16]. As this background is found to be negligible it is ignored in all aspects of the analysis.

**Fig. 2:**
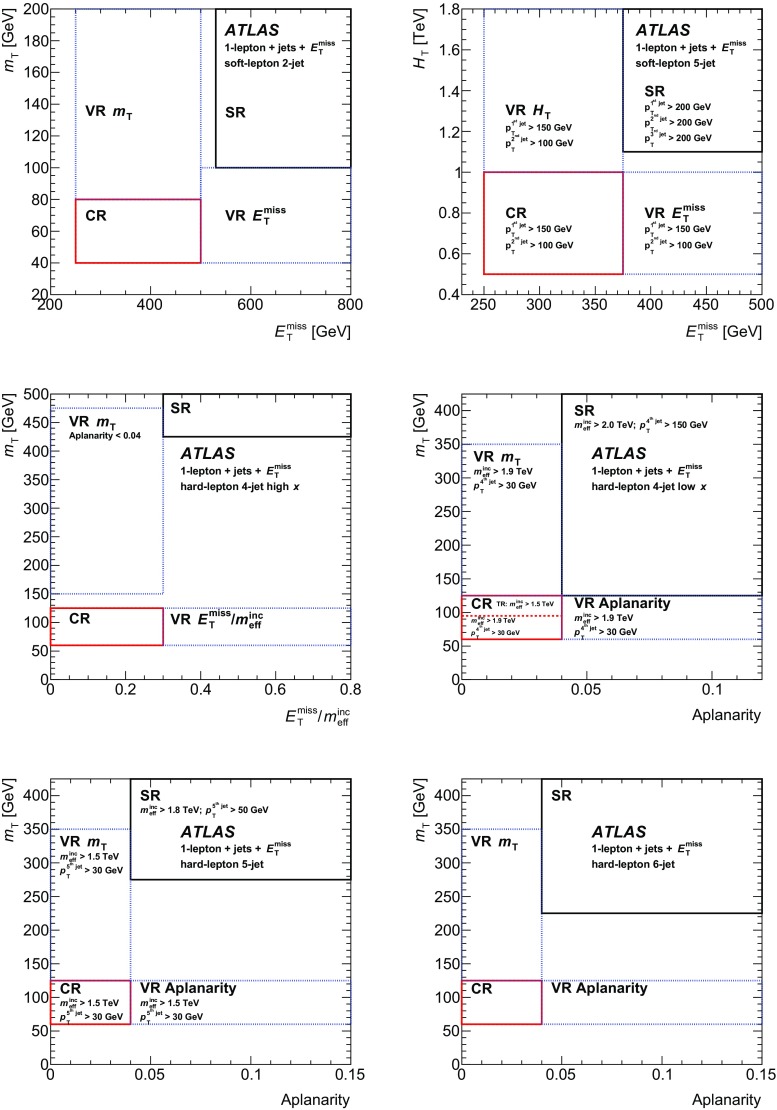
Graphical illustration of the soft-lepton 2-jet (*top left*), soft-lepton 5-jet (*top right*), hard-lepton 4-jet high-*x* (*middle left*), 4-jet low-*x* (*middle right*), 5-jet (*bottom left*) and 6-jet (*bottom right*) signal (SR), control (CR) and validation (VR) regions. In addition to the two variables shown on the *x* and *y* axes, labels indicate other event selections that differ between the corresponding control regions, validation regions and signal regions. The control regions exist in two variants: the $t\bar{t}$ control regions require at least one *b*-tagged jet, while no *b*-tagged jets are required in the *W*+jets control regions

Figure 2 visualises the criteria that define the control regions in the soft-lepton and hard-lepton channels. Based on these, separate control regions are defined to extract the normalisation factors for $t\bar{t}$ and *W*+jets by requiring at least one, or no, *b*-tagged signal jets, respectively. The cross-contamination between these two types of control regions is accounted for in the fit.

**Fig. 3:**
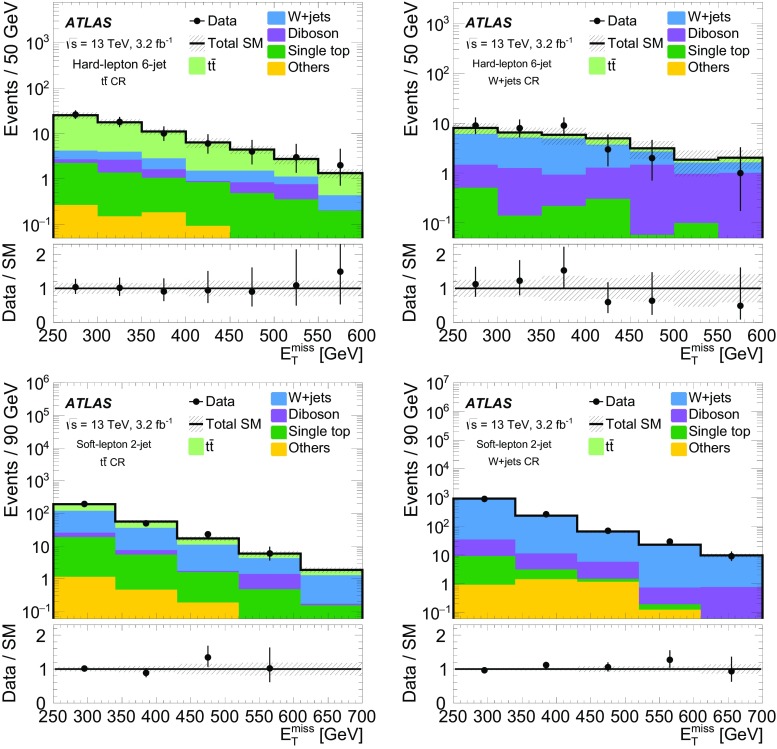
The distribution of the missing transverse momentum is shown in hard-lepton 6-jet $t\bar{t}$ (*top left*) and *W*+jets (*top right*) and in the soft-lepton 2-jet $t\bar{t}$ (*bottom left*) and *W*+jets (*bottom right*) control regions after normalising the $t\bar{t}$ and *W*+jets background processes in the simultaneous fit. In the soft-lepton 2-jet plots, the upper bound on $E_\mathrm{{T}}^\mathrm{{miss}}$ defining the control region is not applied. The *lower panels* of the plots show the ratio of the observed data to the total SM background expected from simulations scaled to the number of events observed in the data. The uncertainty bands include all statistical and systematic uncertainties on simulation, as discussed in Sect. 7. The component ‘Others’ is the sum of *Z*+jets and $t\bar{t}$+V

Figure 3 shows the $E_\mathrm{{T}}^\mathrm{{miss}}$ distribution in selected soft-lepton and hard-lepton control regions. The normalisation of the *W*+jets and $t\bar{t}$ simulations are adjusted to match the observed number of data events in the control region, so that the plots illustrate the modelling of the shape of each variable’s distribution. In general, good agreement between data and background simulations is found within the uncertainties in all the control regions used in the analysis.

To gain confidence in the extrapolation from control to signal regions using simulated event samples, the results of the simultaneous fit are cross-checked in validation regions which are disjoint with both the control and the signal regions. The validation regions are designed to be kinematically close to the signal regions, as shown in Fig. 2, while expecting only a small contamination from the signal in the models considered in this search. The validation regions are not used to constrain parameters in the fit, but they provide a statistically independent cross-check of the extrapolation.

This analysis uses two validation regions per signal region. In the hard-lepton channel, one of the validation regions is used to test the extrapolation to larger $m_{\mathrm {T}}$ values, while the other validation region tests the extrapolation to larger aplanarity values or, in the case of the 4-jet high-*x* selection, to larger values in $E_\mathrm{{T}}^\mathrm{{miss}}$/$m_{\mathrm {eff}}^{\mathrm {incl}}$. In the soft-lepton channel, the validation regions are used to test the extrapolation to larger $E_\mathrm{{T}}^\mathrm{{miss}}$, $m_{\mathrm {T}}$ or $H_{\mathrm {T}}$ values.

## Systematic uncertainties

Two categories of systematic uncertainties have an impact on the results presented here: uncertainties arising from experimental effects and uncertainties associated with theoretical predictions and modelling. Their effects are evaluated for all signal samples and background processes. Since the normalisation of the dominant background processes is extracted in dedicated control regions, the systematic uncertainties only affect the extrapolation to the signal regions in these cases.

Among the dominant experimental systematic uncertainties are the jet energy scale (JES) and resolution (JER) and the muon momentum resolution. The jet uncertainties are derived as a function of $p_{\mathrm {T}}$ and $\eta$ of the jet, as well as of the pile-up conditions and the jet flavour composition of the selected jet sample. They are determined using a combination of simulated samples and studies of data, such as measurements of the jet balance in dijet, *Z*+jet and $\gamma$+jet events [77]. The $J/\psi \rightarrow \ell ^{+}\ell ^{-}$, $W^{\pm }\rightarrow \ell ^{\pm }\nu$ and $Z\rightarrow \ell ^{+}\ell ^{-}$ decays in data and simulation are exploited to estimate the uncertainties in lepton reconstruction, identification, momentum/energy scale and resolution and isolation criteria [69, 70]. In particular, muon momentum resolution and scale calibrations are derived for simulation from a template fit that compares the invariant mass of $Z\rightarrow \mu \mu$ and $J/\psi \rightarrow \mu \mu$ candidates in data and simulation. The corresponding uncertainties are computed from variations of several fit parameters, following the procedure described in Ref. [78].

The simulation is reweighted to match the distribution of the average number of proton-proton interactions per bunch crossing observed in data. In the signal regions characterised by a higher jet multiplicity, the uncertainty arising from this reweighting also becomes relevant.

The systematic uncertainties related to the modelling of $E_\mathrm{{T}}^\mathrm{{miss}}$ in the simulation are estimated by propagating the uncertainties on the energy and momentum scale of each of the objects entering the calculation, as well as the uncertainties on the soft term resolution and scale.

Different uncertainties in the theoretical modelling of the SM production processes are considered, as described in the following.

For $t\bar{t}$, single-top and *W* / *Z*+jets samples, the uncertainties related to the choice of QCD renormalisation and factorisation scales are assessed by varying the corresponding generator parameters up and down by a factor of two around their nominal values. Uncertainties in the resummation scale and the matching scale between matrix elements and parton shower are evaluated for the *W*+jets samples by varying up and down by a factor of two the corresponding parameters in Sherpa .

For $t\bar{t}$ and single-top production, specific samples with an increased and decreased amount of initial- and final-state radiation are compared to the nominal sample. The relative difference in the extrapolation factors ($t\bar{t}$) or expected rates (single top) is assigned as an uncertainty. Moreover, the uncertainty associated with the parton-shower modelling is assessed as the difference between the predictions from powheg+Pythia and powheg+Herwig++ 2 [79].

An uncertainty arising from the choice of parton level generator is estimated for $t\bar{t}$, diboson and *W* / *Z*+jets processes. In the former case, the predictions from powheg-box are compared to aMc@NLO 3 [80]; for dibosons, Sherpa  is compared to powheg-box; for *W* / *Z*+jets, Sherpa  is compared to Madgraph [81].

An uncertainty of 5 % in the inclusive *Z*+jets cross-section is assumed [82]. Uncertainties in the inclusive single-top cross-sections are assigned as 3.7 % (*s*-channel, top), 4.7 % (*s*-channel, anti-top), 4 % (*t*-channel, top), 5 % (*t*-channel, anti-top) and 5.3 % (*Wt*-channel) [83]. Samples using diagram subtraction and diagram removal schemes are compared for assessing the sensitivity to the treatment of interference effects between single-top and $t\bar{t}$ production at NLO.

An overall systematic uncertainty of 6 % in the inclusive cross-section is assigned to the small contribution from *WW*, *WZ*, *ZZ*, $W\gamma$ and $Z\gamma$ processes, which are estimated entirely from simulation. The uncertainty accounts for missing higher-order corrections, for the uncertainty in the value of the strong coupling constant and for the uncertainties on the PDF sets. The uncertainties associated with the resummation, factorisation and renormalisation scales are computed by varying the corresponding Sherpa parameters.

For the very small contributions of $t\bar{t}+W/Z/WW$, an uncertainty of 30 % is assigned.

Among the main systematic uncertainties on the total background predictions in the various signal regions are the ones associated with the finite size of the MC samples, which range from 11 % in the hard-lepton 6-jet SR to 33 % in the hard-lepton 4-jet high-*x* SR. Moreover, the uncertainties associated with the normalisation of the $t\bar{t}$ background, ranging from 7 % in the soft-lepton 5-jet SR to 21 % in the hard-lepton 4-jet low-*x* SR. Further important uncertainties are the theoretical uncertainties associated with the single-top background in the hard-lepton regions, which amount to 3 % in the 4-jet high-*x* signal region and increase to as much as 34 % in the 4-jet low-*x* signal region, and the theoretical uncertainties on the *W*+jets background in the soft-lepton regions (up to 11 %).

The theoretical systematic uncertainty affecting the modelling of ISR can become sizeable in the simplified signal models used in this analysis, especially when the SUSY particles’ mass splitting becomes small. Variations of a factor of two in the following Madgraph and Pythia parameters are used to estimate these uncertainties: the renormalisation and factorisation scales, the initial- and the final-state radiation scales, as well as the Madgraph jet matching scale. The overall uncertainties range from about 5 % for signal models with large mass differences between the gluino, the chargino and the neutralino, to 25 % for models with very compressed mass spectra.

## Statistical analysis

The final results are based on a profile likelihood method [84] using the HistFitter framework [85]. To obtain a set of background predictions that is independent of the observation in the signal regions, the fit can be configured to use only the control regions to constrain the fit parameters; this is referred to as the background-only fit. For each signal region a background-only fit is performed, based on the following inputs:

- the observed number of events in each of the control regions associated with the signal region, together with the number of events expected from simulation;
- the extrapolation factors, including uncertainties, from control regions to the signal region, as obtained from simulation, for the *W*+jets and the $t\bar{t}$ backgrounds;
- the yields of the smaller backgrounds such as the single top, $t\bar{t}$+*V*, *Z*+jets and diboson backgrounds as obtained from simulation, including uncertainties.

Using this information a likelihood is constructed for every background-only fit. It consists of a product of Poisson probability density functions for every region and of constraint terms for systematic uncertainties as described below.

Multiple parameters are included in each likelihood: two normalisation parameters describing the normalisation of the *W*+jets and $t\bar{t}$ backgrounds and nuisance parameters associated with the systematic uncertainties (as described in Sect. 7) or the statistical uncertainties in simulated event yields. The nuisance parameters associated with the systematic uncertainties are constrained by Gaussian functions with their widths corresponding to the size of the uncertainty, while the statistical uncertainties are constrained by Poisson functions. The parameters are correlated between the control regions and the signal region.

In the fit, the likelihood is maximised by adjusting normalisation and nuisance parameters. The normalisation scale factor of the $t\bar{t}$ background is fitted to values between $0.34^{+0.28}_{-0.25}$ (4-jet high-*x* control regions) and $0.92^{+0.14}_{-0.12}$ (5-jet soft-lepton control regions), the normalisation of the *W*+jets background to values between $0.72^{+0.31}_{-0.33}$ (6-jet control regions) and $1.00 \pm 0.04$ (2-jet soft-lepton control regions). Previous analyses [16] also found normalisation factors considerably smaller than unity for these background processes in similarly extreme regions of phase space. The fit introduces correlations between the normalisation parameters associated with the $t\bar{t}$ and the *W*+jets backgrounds and the nuisance parameters associated with systematic uncertainties. The uncertainty in the total background estimate may thus be smaller or larger than the sum in quadrature of the individual uncertainties.

## Results

**Fig. 4:**
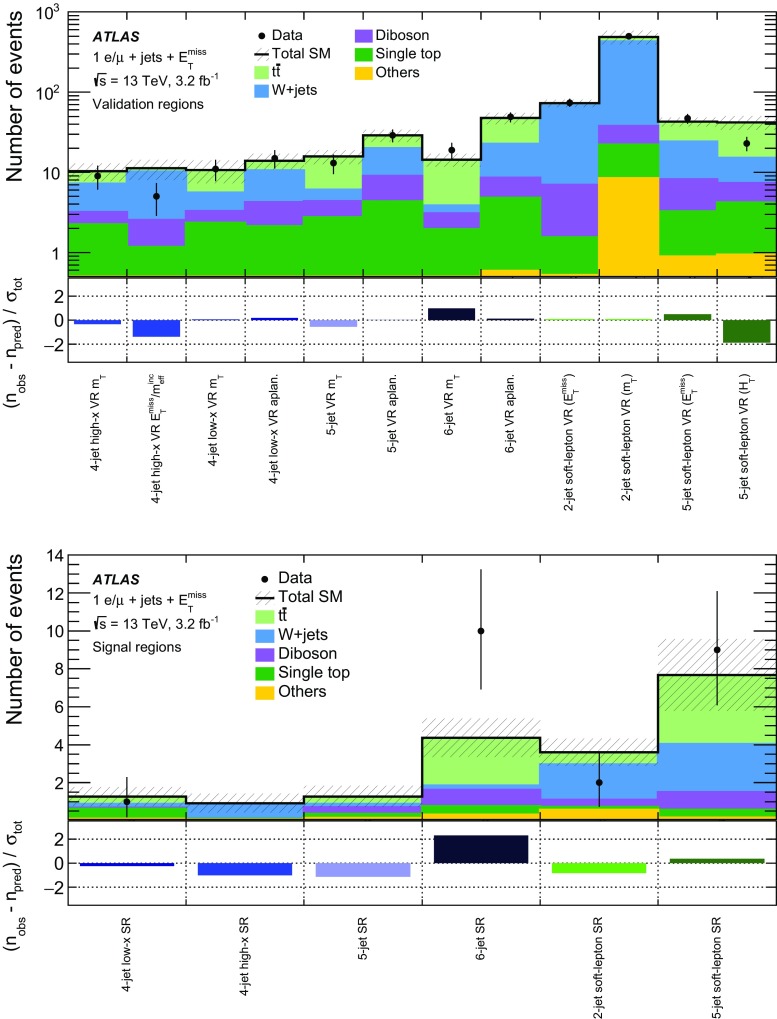
Expected background yields as obtained in the background-only fits in all hard-lepton and soft-lepton validation (*top plot*) and signal (*bottom plot*) regions together with observed data are given in the *top parts* of the plots. Uncertainties in the fitted background estimates combine statistical (in the simulated event yields) and systematic uncertainties. The *bottom parts* of the plots show the differences between observed ($n_\mathrm {obs}$) and predicted $n_\mathrm {pred}$ event yields, divided by the total (statistical and systematic) uncertainty in the prediction ($\sigma _\mathrm {tot}$). *Bars* sharing the *same colour* belong to regions fitted in the same background-only fit

The results of the background-only fit described in Sect. 8 in the validation and signal regions are shown in Fig. 4 and are further detailed for the signal regions in Table 4. Good agreement between predicted and observed event yields is seen in all validation regions.

**Fig. 5:**
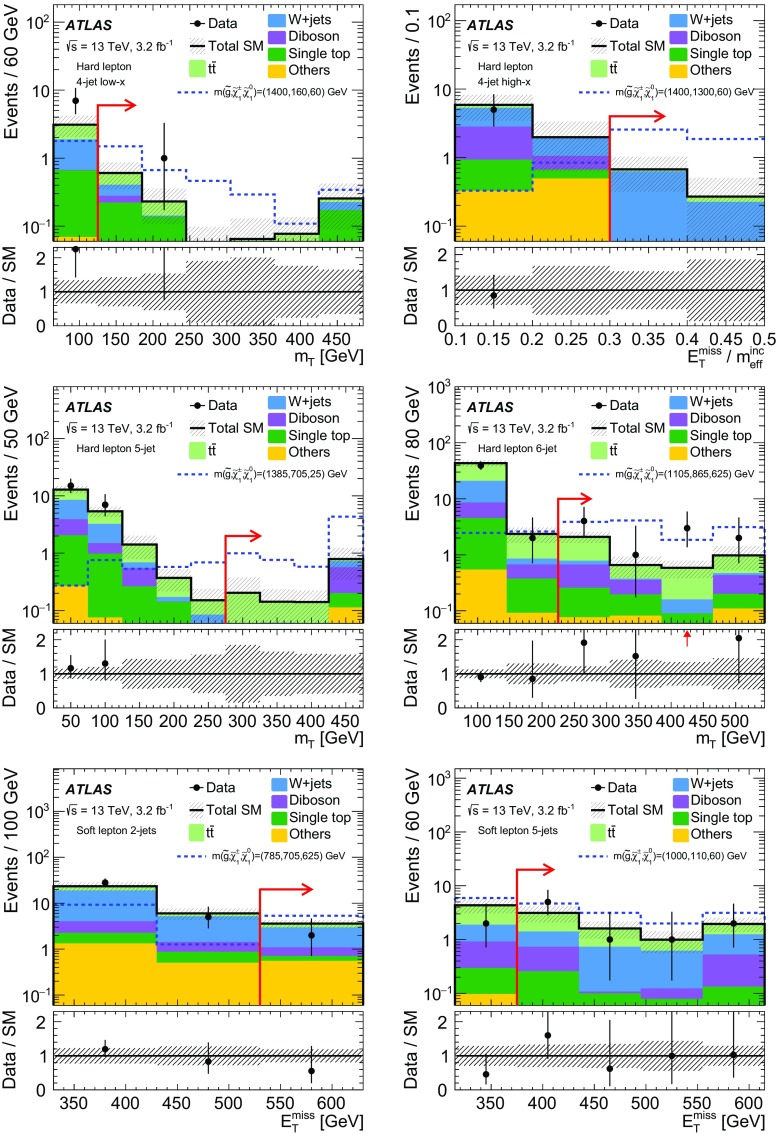
Distributions of $m_{\mathrm {T}}$ for the hard-lepton 4-jet low-*x* (*top left*), 5-jet (*middle left*), 6-jet (*middle right*) signal regions, of $E_\mathrm{{T}}^\mathrm{{miss}}$/$m_{\mathrm {eff}}^{\mathrm {incl}}$ for the 4-jet high-*x* signal region (*top right*) and of $E_\mathrm{{T}}^\mathrm{{miss}}$ for the soft-lepton 2-jet (*bottom left*) and soft-lepton 5-jet (*bottom right*) signal regions. The requirement on the variable plotted is removed from the definitions of the signal regions, where the *arrow* indicates the position of the cut in the signal region. The *lower panels* of the plots show the ratio of the observed data to the total background prediction as derived in the background-only fit. The uncertainty bands plotted include all statistical and systematic uncertainties as discussed in Sect. 7. The component ‘Others’ is the sum of *Z*+jets and $t\bar{t}$+V. The last bin includes the overflow

Figure 5 shows the $m_{\mathrm {T}}$, $E_\mathrm{{T}}^\mathrm{{miss}}$ and $E_\mathrm{{T}}^\mathrm{{miss}}$/$m_{\mathrm {eff}}^{\mathrm {incl}}$ distributions before applying the requirement on the plotted variable in the signal regions.

**Table 4:** Background fit results for the hard-lepton and soft-lepton signal regions, for an integrated luminosity of 3.2 fb$^{-1}$. Uncertainties in the fitted background estimates combine statistical (in the simulated event yields) and systematic uncertainties. The uncertainties in this table are symmetrised for propagation purposes but truncated at zero to remain within the physical boundaries

	Hard-lepton				Soft-lepton	
	4-jet low *x*	4-jet high *x*	5-jet	6-jet	2-jet	5-jet
Observed events	1	0	0	10	2	9
Fitted background events	$1.3 \pm 0.5$	$0.9 \pm 0.5$	$1.3 \pm 0.6$	$4.4 \pm 1.0$	$3.6 \pm 0.7$	$7.7 \pm 1.9$
$t\bar{t}$	$0.40 \pm 0.31$	$0.08 \pm 0.07$	$0.40 \pm 0.24$	$2.5 \pm 0.9$	$0.64 \pm 0.33$	$3.6 \pm 1.2$
*W*+jets	$0.19 \pm 0.12$	$0.8 \pm 0.5$	$0.16 \pm 0.12$	$0.23 \pm 0.16$	$1.9 \pm 0.5$	$2.5 \pm 1.3$
*Z*+jets	$0.045 \pm 0.023$	$0.028 \pm 0.027$	$0.073 \pm 0.035$	$0.08 \pm 0.08$	$0.47 \pm 0.12$	$0.09 \pm 0.04$
Single top	$0.5 \pm 0.5$	$0.04^{+0.10}_{-0.04}$	$0.21^{+0.22}_{-0.21}$	$0.4 \pm 0.4$	$0.16 \pm 0.14$	$0.42 \pm 0.33$
Diboson	$0.06^{+0.20}_{-0.06}$	$0.002^{+0.014}_{-0.002}$	$0.37 \pm 0.23$	$0.9 \pm 0.5$	$0.38 \pm 0.16$	$0.9 \pm 0.6$
$t\bar{t}$+V	$0.048 \pm 0.021$	$0.024 \pm 0.012$	$0.059 \pm 0.029$	$0.23 \pm 0.08$	$0.085 \pm 0.028$	$0.065 \pm 0.024$

The predicted background yields and the observed number of events agree in all signal regions. The largest deviation, 2.1 standard deviations, is observed in the 6-jet hard-lepton signal region. This excess arises only from the muon channel, in which 8 events are observed, while $2.5 \pm 0.7$ events are predicted (local significance of 2.6 standard deviations). The electron channel shows good agreement, with 2 events observed and $1.9 \pm 0.6$ predicted.

Model-independent upper limits and discovery *p* values [85] in the signal regions are calculated in a modified fit configuration with respect to the background-only fit. The only region considered in these fits is the respective signal region. Control regions are not explicitly included and thus any signal contamination in the control regions is not taken into account, thus giving conservative limits. These fits use the background estimates as derived in the background-only fits as input and allow for a non-negative signal contribution in the signal region. An additional normalisation parameter for the signal contribution is included.

Observed and expected upper limits at 95 % confidence level (CL) on the number of events signifying new phenomena beyond the SM ($S_\mathrm {obs}^{95}$ and $S_\mathrm {exp}^{95}$, respectively) are derived based on the $CL_s$ prescription [86] and are shown in Table 5 together with the upper limits on the visible beyond the SM cross-section ($\sigma _\mathrm {vis}$, defined as the product of acceptance, selection efficiency and production cross-section). The latter is calculated by dividing the observed upper limit on the beyond-SM events by the integrated luminosity of 3.2 fb$^{-1}$. The table also gives the background-only confidence level $CL_b$.

Table 5 also shows the discovery *p* values, giving the probability for the background-only assumption to produce event yields greater or equal to the observed data. The $CL_b$ and *p* values use different definitions of test statistics in their calculation, the former with the signal-strength parameter set to one and the latter to zero.

**Table 5:** The columns show from left to right: the name of the respective signal region; the 95 % confidence level (CL) upper limits on the visible cross-section ($\langle \epsilon \sigma \rangle _\mathrm{obs}^{95}$) and on the number of signal events ($S_\mathrm{obs}^{95}$ ); the 95 % CL upper limit on the number of signal events ($S_\mathrm{exp}^{95}$), given the expected number (and $\pm 1\sigma$ variations on the expectation) of background events; the two-sided $CL_b$ value, i.e. the confidence level observed for the background-only hypothesis and the one-sided discovery *p*-value ($p(s = 0)$). The discovery *p* values are capped to 0.5 in the case of observing less events than the fitted background estimates

**Signal region**	$\langle \epsilon \mathrm{\sigma }\rangle _\mathrm{obs}^{95}$[fb]	$S_\mathrm{obs}^{95}$	$S_\mathrm{exp}^{95}$	$CL_{b}$	$p(s=0)$
Hard-lepton					
4-jet low-*x*	1.23	3.9	$$ { 4.1 }^{ +1.5 }_{ -0.9 }$$	0.46	0.50
4-jet high-*x*	0.87	2.8	$$ { 2.9 }^{ +1.3 }_{ -0.2 }$$	0.27	0.50
5-jet	0.87	2.8	$$ { 3.5 }^{ +1.4 }_{ -0.7 }$$	0.19	0.50
6-jet	3.90	12.5	$$ { 6.5 }^{ +2.6 }_{ -1.6 }$$	0.98	0.02
Soft-lepton					
2-jet	1.33	4.3	$$ { 5.3 }^{ +2.2 }_{ -1.3 }$$	0.23	0.50
5-jet	2.87	9.2	$$ { 8.1 }^{ +2.9 }_{ -2.1 }$$	0.68	0.34

**Fig. 6:**
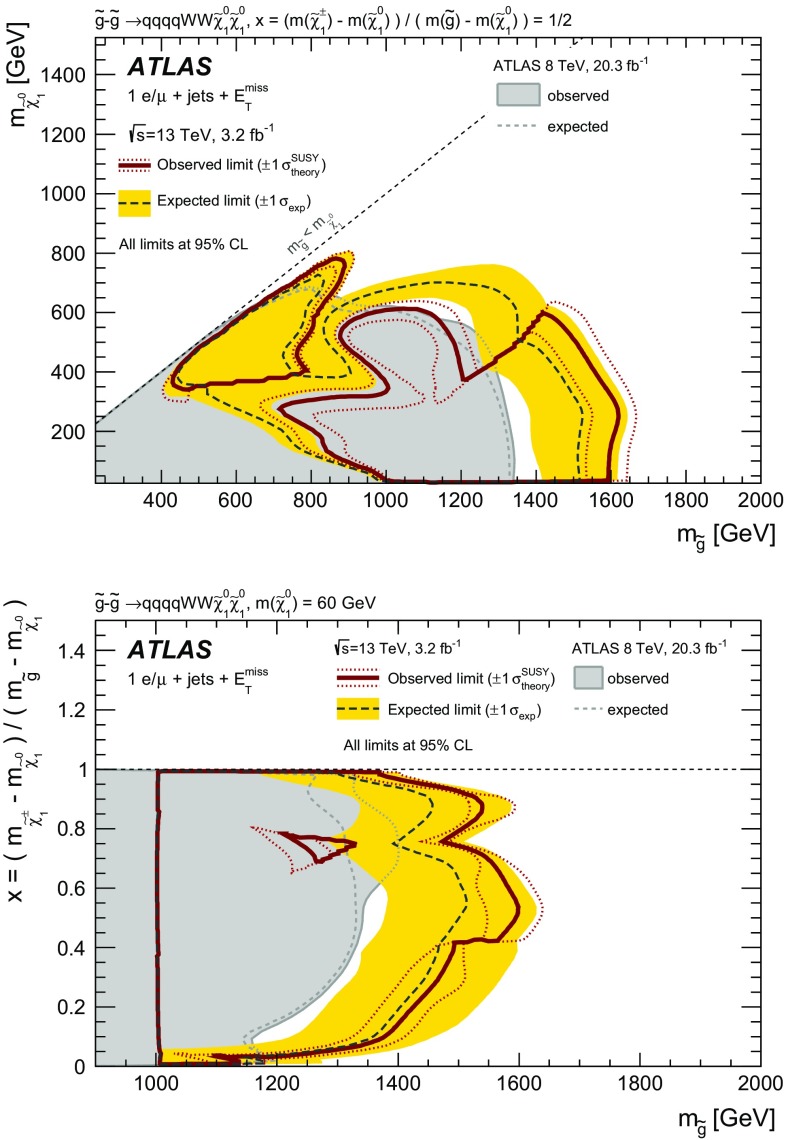
Combined 95 % CL exclusion limits in the two gluino simplified models using for each model point the signal region with the best expected sensitivity. The limits are presented in the ($m_{\tilde{g}},m_{\tilde{\chi }^{0}_{1}}$) mass plane (*top*) for the scenario where the mass of the chargino $\tilde{\chi }_{1}^{\pm }$ is fixed to $x = (m_{\tilde{\chi }_{1}^{\pm }} - m_{\tilde{\chi }^{0}_{1}})/(m_{\tilde{g}} - m_{\tilde{\chi }^{0}_{1}}) = 1/2$ and in the ($m_{\tilde{g}},x$) plane (*bottom*) for the $m_{\tilde{\chi }^{0}_{1}} = 60 \mathrm {\ Ge V}$ models. The *red solid line* corresponds to the observed limit with the *red dotted lines* indicating the $\pm 1 \sigma$ variation of this limit due to the effect of theoretical scale and PDF uncertainties in the signal cross-section. The *dark grey dashed line* indicates the expected limit with the *yellow band* representing the $\pm 1 \sigma$ variation of the median expected limit due to the experimental and theoretical uncertainties. The exclusion limits at 95 % CL by previous ATLAS analyses [17] are shown as the *grey area*

Model-dependent limits are calculated in a modified fit configuration with respect to the background-only fit. A signal contribution is allowed and considered in all control and signal regions, with a non-negative signal-strength normalisation parameter included. For the signal processes, uncertainties due to detector effects and theoretical modelling are considered. The signal regions are explicitly used in the fit to constrain the likelihood parameters. Figure 6 shows the combined 95 % CL exclusion limits in the simplified models with gluino production using for each model point the signal region with the best expected sensitivity. Gluino masses up to 1.6 Te V are excluded for scenarios with large mass differences between the gluino and the neutralino and $x = (m_{\tilde{\chi }_{1}^{\pm }} - m_{\tilde{\chi }^{0}_{1}})/(m_{\tilde{g}} - m_{\tilde{\chi }^{0}_{1}}) = 1/2$. In the same scenario and for models with a small mass difference between the gluino and the neutralino, gluino masses up to 870 Ge V are excluded. The signal regions address very different sets of models and are complementary to each other. In the case of the hard-lepton 6-jet signal region (covering the central part in the ($m_{\tilde{g}},m_{\tilde{\chi }^{0}_{1}}$) mass plane in Fig. 6), the observed exclusion limit is considerably weaker than the expected one due to the excess seen in this region.

## Conclusion

A search for gluinos in events with one isolated lepton, jets and missing transverse momentum is presented. The analysis uses 3.2 fb$^{-1}$of proton–proton collision data collected by the ATLAS experiment in 2015 at $\sqrt{s} = 13 \mathrm{{\ Te V}}$ at the LHC. Six signal regions requiring at least two to six jets are used to cover a broad spectrum of the targeted SUSY model parameter space. While four signal regions are based on high-$p_{\mathrm {T}}$ lepton selections and target models with large mass differences between the supersymmetric particles, two dedicated low-$p_{\mathrm {T}}$ lepton regions are designed to enhance the sensitivity to models with compressed mass spectra.

The observed data agree with the Standard Model background prediction in the signal regions. The largest deviation is a 2.1 standard deviation excess in a channel requiring a high-$p_{\mathrm {T}}$ lepton and six jets. For all signal regions, limits on the visible cross-section are derived in models of new physics within the kinematic requirements of this search. In addition, exclusion limits are placed on models with gluino production and subsequent decays via an intermediate chargino to the lightest neutralino. The exclusion limits of previous searches conducted in LHC Run 1 are significantly extended. Gluino masses up to $1.6~\mathrm{{\ Te V}}$ are excluded for low neutralino masses (${\lesssim }{300}\mathrm {\ Ge V}$) and chargino masses of ${\sim }850 \mathrm {\ Ge V}$.

## Footnotes

1 ATLAS uses a right-handed coordinate system with its origin at the nominal interaction point (IP) in the centre of the detector and the *z*-axis along the beam pipe. The *x*-axis points from the IP to the centre of the LHC ring, and the *y*-axis points upward. Cylindrical coordinates $(r,\phi )$ are used in the transverse plane, $\phi$ being the azimuthal angle around the *z*-axis. The pseudorapidity is defined in terms of the polar angle $\theta$ as $\eta =-\ln \tan (\theta /2)$ and the rapidity is defined as $y = 0.5 \ln [(E + p_z)/(E - p_z)]$, where *E* is the energy and $p_z$ the longitudinal momentum of the object of interest.

2 The Herwig++ version 2.7.1 used.

3 The aMc@NLO version 2.1.1 is used.

## References

[1] Yu. A. Golfand, E.P. Likhtman, Extension of the Algebra of Poincare Group generators and violation of p invariance, JETP Lett. **13**, 323–326 (1971) [Pisma Zh. Eksp. Teor. Fiz.13,452(1971)]

[2] Volkov DV, Akulov VP (1973). Is the Neutrino a Goldstone Particle?. Phys. Lett. B.

[3] Wess J, Zumino B (1974). Supergauge transformations in four-dimensions. Nucl. Phys. B.

[4] Wess J, Zumino B (1974). Supergauge invariant extension of quantum electrodynamics. Nucl. Phys. B.

[5] Ferrara S, Zumino B (1974). Supergauge invariant Yang-Mills theories. Nucl. Phys. B.

[6] Salam A, Strathdee JA (1974). Supersymmetry and Nonabelian gauges. Phys. Lett. B.

[7] Fayet P (1976). Supersymmetry and weak, electromagnetic and strong interactions. Phys. Lett. B.

[8] Fayet P (1977). Spontaneously broken supersymmetric theories of weak, electromagnetic and strong interactions. Phys. Lett. B.

[9] Sakai N (1981). Naturalness in supersymmetric guts. Z. Phys. C.

[10] Dimopoulos S, Raby S, Wilczek F (1981). Supersymmetry and the scale of unification. Phys. Rev. D.

[11] Ibañez LE, Ross GG (1981). Low-energy predictions in supersymmetric grand unified theories. Phys. Lett. B.

[12] Dimopoulos S, Georgi H (1981). Softly broken supersymmetry and SU(5). Nucl. Phys. B.

[13] Farrar GR, Fayet P (1978). Phenomenology of the production, decay, and detection of new Hadronic States associated with supersymmetry. Phys. Lett. B.

[14] H. Goldberg, Constraint on the photino mass from cosmology, Phys. Rev. Lett. **50**, 1419 (1983) [Erratum: Phys. Rev. Lett.103,099905(2009)]

[15] Ellis JR (1984). Supersymmetric relics from the big bang. Nucl. Phys. B.

[16] ATLAS Collaboration, Search for squarks and gluinos in events with isolated leptons, jets and missing transverse momentum at $$\sqrt{s} = 8$$ TeV with the ATLAS detector, JHEP **04**, 116 (2015). arXiv:1501.03555 [hep-ex]

[17] ATLAS Collaboration, Summary of the searches for squarks and gluinos using $$\sqrt{s}=8$$ TeV pp collisions with the ATLAS experiment at the LHC, JHEP **10**, 054 (2015). arXiv:1507.05525 [hep-ex]

[18] CMS Collaboration, Search for new physics in events with same-sign dileptons and jets in pp collisions at $$\sqrt{s} = 8$$ TeV, JHEP **01**, 163 (2014) [Erratum: JHEP01,014(2015)]. arXiv:1311.6736

[19] C.M.S. Collaboration, Search for new physics in the multijet and missing transverse momentum final state in proton-proton collisions at $$\sqrt{s}= 8$$ TeV. JHEP **06**, 055 (2014). arXiv:1402.4770 [hep-ex] 10.1103/PhysRevLett.109.171803 23215177

[20] CMS Collaboration, Search for supersymmetry in events with one lepton in proton-proton collisions at $$\sqrt{s}=13$$ TeV with the CMS experiment (2016). https://cds.cern.ch/record/2140638

[21] ATLAS Collaboration, The ATLAS experiment at the CERN Large Hadron Collider, JINST **3**, S08003 (2008)

[22] ATLAS Collaboration, ATLAS insertable B-layer technical design report, ATLAS-TDR-19 (2010). http://cds.cern.ch/record/1291633

[23] ATLAS Collaboration, Performance of the ATLAS trigger system in 2010, Eur. Phys. J. C **72**, 1849 (2012). arXiv:1110.1530 [hep-ex]

[24] J. Alwall et al., Searching for directly decaying Gluinos at the Tevatron. Phys. Lett. B **666**, 34–37 (2008). arXiv:0803.0019 [hep-ph]

[25] J. Alwall, P. Schuster, N. Toro, Simplified models for a first characterization of new physics at the LHC. Phys. Rev. D **79**, 075020 (2009). arXiv:0810.3921 [hep-ph]

[26] D. Alves et al., Simplified models for LHC new physics searches. J. Phys. G: Nucl. Part. Phys. **39**, 105005 (2012). arXiv:1105.2838 [hep-ph]

[27] J. Alwall et al., The automated computation of tree-level and next-to-leading order differential cross sections, and their matching to parton shower simulations. JHEP **07**, 079 (2014). arXiv:1405.0301 [hep-ph]

[28] T. Sjöstrand, S. Mrenna, P.Z. Skands, A. Brief, Introduction to PYTHIA 8.1. Comput. Phys. Commun. **178**, 852–867 (2008). arXiv:0710.3820 [hep-ph]

[29] L. Lönnblad, S. Prestel, Matching tree-level matrix elements with interleaved showers. JHEP **03**, 019 (2012). arXiv:1109.4829 [hep-ph]

[30] ATLAS Collaboration, ATLAS Pythia 8 tunes to 7 TeV data, ATL-PHYS-PUB-2014-021 (2014). http://cdsweb.cern.ch/record/1966419

[31] R.D. Ball et al., Parton distributions with LHC data. Nucl. Phys. B **867**, 244–289 (2013). arXiv:1207.1303 [hep-ph]

[32] Lange DJ (2001). The EvtGen particle decay simulation package. Nucl. Instrum. Methods A.

[33] W. Beenakker et al., Squark and gluino production at hadron colliders, Nucl. Phys. B **492**, 51–103 (1997). arXiv:hep-ph/9610490 [hep-ph]

[34] A. Kulesza, L. Motyka, Threshold resummation for squark-antisquark and gluino-pair production at the LHC. Phys. Rev. Lett. **102**, 111802 (2009). arXiv:0807.2405 [hep-ph] 10.1103/PhysRevLett.102.111802 19392192

[35] A. Kulesza, L. Motyka, Soft gluon resummation for the production of gluino-gluino and squark-antisquark pairs at the LHC. Phys. Rev. D **80**, 095004 (2009). arXiv:0905.4749 [hep-ph] 10.1103/PhysRevLett.102.111802 19392192

[36] W. Beenakker et al., Soft-gluon resummation for squark and gluino hadroproduction. JHEP **12**, 041 (2009). arXiv:0909.4418 [hep-ph]

[37] W. Beenakker et al., Squark and gluino hadroproduction. Int. J. Mod. Phys. A **26**, 2637–2664 (2011). arXiv:1105.1110 [hep-ph]

[38] M. Krämer et al., Supersymmetry production cross sections in pp collisions at $$\sqrt{s} = 7$$ TeV (2012). arXiv:1206.2892 [hep-ph]

[39] C. Borschensky et al., Squark and gluino production cross sections in pp collisions at $$\sqrt{s} = 13, 14, 33$$ and 100 TeV. Eur. Phys. J. C **74**, 3174 (2014). arXiv:1407.5066 [hep-ph] 10.1140/epjc/s10052-014-3174-y PMC4423871 25983637

[40] T. Gleisberg et al., Event generation with SHERPA 1.1, JHEP **02**, 007 (2009). arXiv:0811.4622 [hep-ph]

[41] T. Sjöstrand, S. Mrenna, P.Z. Skands, PYTHIA 6.4 Physics and Manual, JHEP **05**, 026 (2006). arXiv:hep-ph/0603175

[42] P.Z. Skands, Tuning Monte Carlo generators: the Perugia tunes. Phys. Rev. D **82**, 074018 (2010). arXiv:1005.3457 [hep-ph]

[43] ATLAS Collaboration, Simulation of top quark production for the ATLAS experiment at $$\sqrt{s} = 13$$ TeV. (2016). http://cds.cern.ch/record/2120417

[44] S. Alioli et al., A general framework for implementing NLO calculations in shower Monte Carlo programs: the POWHEG BOX. JHEP **06**, 043 (2010). arXiv:1002.2581 [hep-ph]

[45] H.-L. Lai et al., New parton distributions for collider physics. Phys. Rev. D **82**, 074024 (2010). arXiv:1007.2241 [hep-ph]

[46] P. Artoisenet et al., Automatic spin-entangled decays of heavy resonances in Monte Carlo simulations. JHEP **03**, 015 (2013). arXiv:1212.3460 [hep-ph]

[47] J. Pumplin et al., New generation of parton distributions with uncertainties from global QCD analysis. JHEP **07**, 012 (2002). arXiv:hep-ph/0201195 [hep-ph]

[48] ATLAS Collaboration, Monte Carlo generators for the production of a $$W$$ or $$Z/\gamma ^{\ast }$$ Boson in association with jets at ATLAS in Run 2 (2016). http://cds.cern.ch/record/2120133

[49] T. Gleisberg, S. Höche, Comix, a new matrix element generator. JHEP **12**, 039 (2008). arXiv:0808.3674 [hep-ph]

[50] F. Cascioli, P. Maierhofer, S. Pozzorini, Scattering amplitudes with open loops. Phys. Rev. Lett. **108**, 111601 (2012). arXiv:1111.5206 [hep-ph] 10.1103/PhysRevLett.108.111601 22540459

[51] S. Schumann, F. Krauss, A Parton shower algorithm based on Catani-Seymour dipole factorisation. JHEP **03**, 038 (2008). arXiv:0709.1027 [hep-ph]

[52] S. Höche et al., QCD matrix elements + parton showers: the NLO case. JHEP **04**, 027 (2013). arXiv:1207.5030 [hep-ph]

[53] ATLAS Collaboration, Single Boson and Diboson production cross sections in pp collisions at $$\sqrt{s} = 7$$ TeV (2010). https://cds.cern.ch/record/1287902

[54] ATLAS Collaboration, Multi-Boson simulation for 13 TeV ATLAS analyses (2016). http://cds.cern.ch/record/2119986

[55] ATLAS Collaboration, Modelling of the $$t\bar{t}H$$ and $$t\bar{t}V (V = W, Z)$$ processes for $$\sqrt{s} = 13$$ TeV ATLAS analyses (2016). http://cds.cern.ch/record/2120826

[56] A. Lazopoulos et al., Next-to-leading order QCD corrections to $$t-\bar{t}- Z$$ production at the LHC. Phys. Lett. B **666**, 62 (2008). arXiv:hep-ph/0804.2220 [hep-ph]

[57] J.M. Campbell, R.K. Ellis, $$t\bar{t}$$ W production and decay at NLO. JHEP **07**, 052 (2012). arXiv:hep-ph/1204.5678 [hep-ph]

[58] ATLAS Collaboration, The ATLAS simulation infrastructure, Eur. Phys. J. C **70**, 823–874 (2010). arXiv:1005.4568 [physics.ins-det]

[59] GEANT4 Collaboration (2003). GEANT4: a simulation toolkit. Nucl. Instrum. Meth. A.

[60] ATLAS Collaboration, The simulation principle and performance of the ATLAS fast calorimeter simulation FastCaloSim, ATL-PHYS-PUB-2010-013 (2010). http://cds.cern.ch/record/1300517

[61] A. Sherstnev, R. Thorne, Parton distributions for LO generators. Eur. Phys. J. C **55**, 553–575 (2008). arXiv:0711.2473 [hep-ph]

[62] ATLAS Collaboration, Summary of ATLAS Pythia 8 tunes, ATL-PHYS-PUB-2012-003 (2012). http://cds.cern.ch/record/1474107

[63] ATLAS Collaboration, Improved luminosity determination in pp collisions at $$\sqrt{s} = 7$$ TeV using the ATLAS detector at the LHC, Eur. Phys. J. C **73**, 2518 (2013). arXiv:1302.4393 [hep-ex] 10.1140/epjc/s10052-013-2518-3 PMC4370906 25814867

[64] M. Cacciari, G.P. Salam, G. Soyez, The Anti-k(t) jet clustering algorithm. JHEP **04**, 063 (2008). arXiv:0802.1189 [hep-ph]

[65] ATLAS Collaboration, Properties of jets and inputs to jet reconstruction and calibration with the ATLAS detector using proton-proton collisions at $$\sqrt{s} = 13$$ TeV (2015). http://cds.cern.ch/record/2044564

[66] Cacciari M, Salam GP (2008). Pileup subtraction using jet areas. Phys. Lett. B.

[67] ATLAS Collaboration, Performance of pile-up mitigation techniques for jets in $$pp$$ collisions at $$\sqrt{s} = 8$$ TeV using the ATLAS detector (2015). arXiv:1510.03823 [hep-ex]

[68] ATLAS Collaboration, Selection of jets produced in 13 TeV proton-proton collisions with the ATLAS detector (2015). http://cds.cern.ch/record/2037702

[69] ATLAS Collaboration, Electron identification measurements in ATLAS using $$\sqrt{s} = 13$$ TeV data with 50 ns bunch spacing (2015). http://cds.cern.ch/record/2048202

[70] ATLAS Collaboration, Muon reconstruction performance in early $$\sqrt{s} = 13$$ TeV data (2015). http://cds.cern.ch/record/2047831

[71] ATLAS Collaboration, Expected performance of missing transverse momentum reconstruction for the ATLAS detector at $$\sqrt{s} = 13$$ TeV (2015). http://cds.cern.ch/record/2037700

[72] ATLAS Collaboration, Performance of missing transverse momentum reconstruction for the ATLAS detector in the first proton-proton collisions at at $$\sqrt{s}= 13$$ TeV (2015). http://cds.cern.ch/record/2037904 10.1140/epjc/s10052-018-6288-9 PMC6394290 30880822

[73] ATLAS Collaboration, Tagging and suppression of pileup jets with the ATLAS detector (2014). http://cds.cern.ch/record/1700870

[74] ATLAS Collaboration, Expected performance of the ATLAS b-tagging algorithms in Run-2 (2015). http://cds.cern.ch/record/2037697

[75] ATLAS Collaboration, Commissioning of the ATLAS b-tagging algorithms using $$t\bar{t}$$ events in early Run-2 data (2015). http://cds.cern.ch/record/2047871

[76] C. Chen, New approach to identifying boosted hadronically decaying particles using jet substracture in its center-of-mass frame. Phys. Rev. D **85**, 034007 (2012). arXiv:1112.2567 [hep-ph]

[77] ATLAS Collaboration, Jet calibration and systematic uncertainties for jets reconstructed in the ATLAS detector at $$\sqrt{s} = 13$$ TeV (2015). http://cds.cern.ch/record/2037613

[78] ATLAS Collaboration, Measurement of the muon reconstruction performance of the ATLAS detector using 2011 and 2012 LHC proton-proton collision data, Eur. Phys. J. C **74**, 3130 (2014). arXiv:1407.3935 [hep-ex] 10.1140/epjc/s10052-014-3130-x PMC4371046 25814875

[79] M. Bahr et al., Herwig++ Physics and Manual. Eur. Phys. J. C **58**, 639–707 (2008). arXiv:0803.0883 [hep-ph]

[80] S. Frixione, B.R. Webber, Matching NLO QCD computations and parton shower simulations. JHEP **06**, 029 (2002). arXiv:hep-ph/0204244 [hep-ph]

[81] J. Alwall et al., MadGraph 5: going beyond. JHEP **06**, 128 (2011). arXiv:1106.0522 [hep-ph]

[82] ATLAS Collaboration, Measurement of W and Z Boson production cross sections in pp collisions at root $$\sqrt{s} = 13$$ TeV in the ATLAS detector (2015). http://cds.cern.ch/record/2045487

[83] P. Kant et al., HatHor for single top-quark production: Updated predictions and uncertainty estimates for single top-quark production in hadronic collisions. Comput. Phys. Commun. **191**, 74–89 (2015). arXiv:1406.4403 [hep-ph]

[84] G. Cowan et al., Asymptotic formulae for likelihood-based tests of new physics, Eur. Phys. J. C **71**, 1554 (2011). arXiv:1007.1727 [physics.data-an]

[85] M. Baak et al., HistFitter software framework for statistical data analysis. Eur. Phys. J. C **75**, 153 (2015). arXiv:1410.1280 [hep-ex]

[86] Read AL (2002). Presentation of search results: The CL(s) technique. J. Phys. G.

[87] ATLAS Collaboration, ATLAS computing acknowledgements 2016–2017, ATL-GEN-PUB-2016-002, http://cds.cern.ch/record/2202407

